# The plastics integrated assessment model (PLAIA): Assessing emission mitigation pathways and circular economy strategies for the plastics sector^☆^

**DOI:** 10.1016/j.mex.2022.101666

**Published:** 2022-03-15

**Authors:** Paul Stegmann, Vassilis Daioglou, Marc Londo, Martin Junginger

**Affiliations:** a Utrecht University, Utrecht, the Netherlands; b PBL Netherlands Environmental Assessment Agency, Den Haag, the Netherlands; c Netherlands Association for Renewable Energy (NVDE), Utrecht, the Netherlands

**Keywords:** Plastics, Climate change, Circular economy, Recycling, Waste, Waste management, Bioeconomy, Biomass, Greenhouse-gas emissions, Integrated assessment modeling, GDP, Gross domestic product, HDPE, High density polyethylene, IAM, Integrated assessment model, IMAGE, An Integrated Model to Assess the Global Environment, IPCC, Intergovernmental Panel on Climate Change, LD,LDPE, (Linear) low density polyethylene, NEDE, Non-energy demand and emissions model, PET, Polyethylene terephthalate, PLAIA, Plastics Integrated Assessment model, PP, Polypropylene, PP&A, Polyester, polyamide, and acrylic, PS, Polystyrene, PUR, Polyurethanes, PVC, Polyvinylchloride, SSP, Shared socioeconomic pathway, TIMER, Energy model of the IMAGE framework, WTO, Waste Treatment Option

## Abstract

Integrated assessment models (IAM) study the interlinkages between human and natural systems and play a key role in assessing global strategies to reduce global warming. However, they largely neglect the role of materials and the circular economy. With the Plastics Integrated Assessment model (PLAIA), we included plastic production, use, and end-of-life in the IAM IMAGE. PLAIA models the global plastics sector and its impacts up to 2100 for 26 world regions, providing a long-term, dynamic perspective of the sector and its interactions with other socioeconomic and natural systems. This article summarizes the model structure, mathematical formulation, assumptions, and data sources. The model links the upstream chemical production with the downstream production of plastics, their use in different sectors, and their end of life. Therefore, PLAIA can assess material use and emission mitigation strategies throughout the whole life cycle in an IAM, including the impacts of the circular economy on mitigating climate change. PLAIA projects plastics demand, production pathways and specifies the annual plastic waste generation, collection, and the impact of waste management strategies. It shows the fossil and bio-based energy and carbon flows in product stocks, landfills, and the emissions in production and at the end of life.

• We included plastics production, use, and waste management into an Integrated Assessment Model (IAM).
• Our model PLAIA provides a long-term, dynamic perspective of the global plastics sector until 2100 and its interactions with other sectors and the environment.
• PLAIA can assess the impact of material use and emission mitigation strategies throughout the whole life cycle of plastics.

## Graphical abstract

**Figure:**
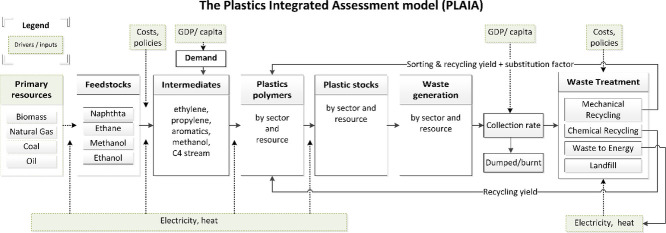

**Specifications Table**

**Table:** 

Subject Area	Environmental Science
More specific subject area:	*Integrated assessment modeling of climate change impacts*
Method name:	Plastics integrated assessment model (PLAIA)
Name and reference of original method:	Daioglou, Vassilis, Andre P. C. Faaij, Deger Saygin, Martin K. Patel, Birka Wicke, and Detlef P. van Vuuren. 2014. “Energy Demand and Emissions of the Non-Energy Sector.” *Energy Environ. Sci.* 7(2):482–98. Stehfest, E., D. van Vuuren, T. Kram, A. F. Bouwman, R. Alkemade, M. Bakkenes, H. Biemans, A. F. Bouwman, M. den Elzen, J. Janse, P. L. Lucas, J. G. Van Minnen, C. Müller, and A. G. Prins. 2014. *Integrated Assessment of Global Environmental Change with IMAGE 3.0 - Model Description and Policy Applications*. The Hague.
Resource availability:	https://www.pbl.nl/en/image/about-image

## Overview of the model

### Purpose of the model

Plastics show the fastest-growing production of all bulk materials globally - with considerable sustainability implications. The plastics sector caused 4.5% of global Greenhouse-gas (GHG) emissions in 2015 [3]. GHG emissions of the sector could quadruple until 2050 with current growth rates in production [64]. Moreover, plastics pose a significant challenge once they become waste, contributing to global plastic pollution [30]. Yet, remarkably few model studies have dealt with scenarios for plastics and response strategies on a global scale until 2050 and beyond.

We are the first to add the plastics sector and circular economy strategies to one of the leading global energy, land, and emissions integrated assessment models the Intergovernmental Panel on Climate Change (IPCC) uses to analyze the pathways to achieve the Paris climate targets. Our Plastics Integrated Assessment (PLAIA) model is designed to assess the entire life cycle of plastics globally and for 26 world regions, making it one of the first models of the plastics sector that allow for a global and regional analysis. Moreover, it is the first plastics model that assesses the long-term dynamics of the plastics sector until 2100.

The PLAIA model allows the assessment of different strategies in the production, use, and waste management of plastics and how they affect the sector's material flows, energy use, and emissions. Examples of such strategies include changes in plastic demand, renewable energy use, feedstock substitution in plastic production (e.g., biomass instead of coal and oil), product lifetime extension, reuse, and recycling. As part of an integrated assessment model (IAM), PLAIA also provides insights into the interactions of the plastics sector with the energy and agricultural sectors as well as with the climate, water, and land systems (see the following section).

Key questions this model is designed to answer include:

• Quantifying the material and energy use of the plastics sector and the corresponding GHG emissions for different scenarios.
• Quantifying the impact of different GHG mitigation strategies for the plastics sector on its material and energy use and GHG emissions.
• Quantifying the impact of circular economy strategies for the plastics sector on its material and energy use and GHG emissions.
• Analyzing the impact of other economic sectors and natural systems on the plastics sector and vice versa.
• Analyzing trade-offs between sustainability targets, e.g., between climate and circular economy targets, or climate and land use.

This article describes the structure, mathematical formulation, data sources, and assumptions of the PLAIA model and discusses its key limitations. It is supposed to complement accompanying articles that discuss the model's results for different scenarios. Such articles are currently under review or in preparation [52]. The purpose of this publication is to support other researchers in developing similar models of the plastics sector and to provide methodological details to interested readers of our other publications based on the PLAIA model.

### Integrated assessment models (IAM)

Integrated assessment models (IAM) study the interlinkages between human and natural systems and their impact on the world's climate [50]. They consist of several sub-models covering various natural systems like land, water, biodiversity, and human systems like energy use and agriculture [58]. They have played a key role in assessing strategies to mitigate climate change [56] such as in the reports of the Intergovernmental Panel on Climate Change (IPCC) [46]. Concerning the energy system, IAMs traditionally neglect non-energy uses of energy resources, i.e., their use for chemicals and plastics. Consequentially, they also do not cover the fate of the energy resources bound in chemicals and plastics at their end of life and the corresponding emissions.

The IMAGE model [37] is one of the few IAMs which offers a sub-model for non-energy use and emissions (the NEDE model), incorporating the key outputs of the chemical sector on an aggregated level [7]. However, this model focuses on the upstream chemical production, limiting its use for assessing downstream resource efficiency measures, i.e., on the consumer side or at the end of life of products. Furthermore, it does not cover the production, use, and end-of-life of plastics products.

This article introduces the first global plastics production & waste management models as part of an IAM, namely the Plastics Integrated Assessment model (PLAIA). PLAIA can be considered an add-on to the NEDE model, allowing for assessing downstream material use and emission mitigation strategies. By assessing plastic production and waste management in an IAM, we can offer a dynamic, long-term perspective that highlights the sector's interaction with other socioeconomic and natural systems.

### Non-energy demand and emissions (NEDE) model

**Fig. 1:**
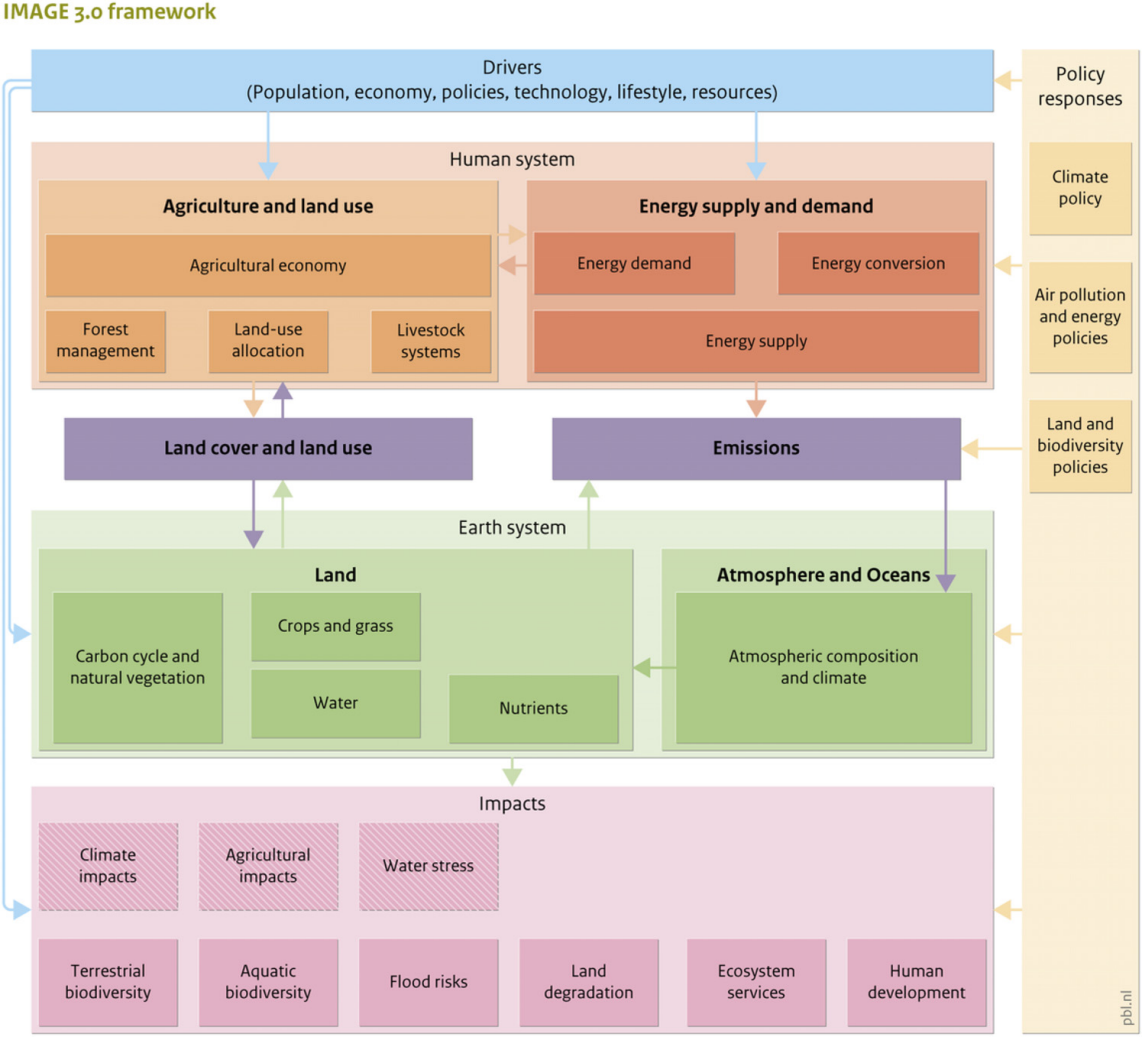
The framework of the IMAGE model. For a detailed explanation of IMAGE see PBL [37] and Stehfest et al. [53].

The NEDE model was developed to assess trends in energy and feedstock use and explore possible mitigation strategies for the chemical sector until the year 2100 [7]. NEDE is embedded in the TIMER model, a recursive dynamic simulation model of the energy system and part of the integrated assessment model IMAGE [8]. The model assesses developments for 26 world regions (see Figure 13 and Table 1 in the supplementary materials). The IMAGE model structure is displayed in Fig. 1 and described in PBL [37] and Stehfest et al. [53].

NEDE is driven by the product demand for high value chemicals (HVC)^1^, ammonia, methanol and refinery products^2^, which are determined by assumptions on population and economic growth (GDP/capita), e.g., based on the shared socioeconomic pathways (SSP) [34]. This demand can be met by different technology pathways, based on the primary energy carriers coal, oil, natural gas, and biomass. The primary energy demand of each carrier (expressed in GJ) is calculated based on the conversion efficiencies of the technology pathways and their respective market shares [7].

In its original version of [7,8], NEDE models the chemical sector very aggregately, and its scope is limited to the upstream production of intermediates like ethylene and aromatics, thus excluding the downstream production steps and end-products. A reason for this aggregated approach with an upstream focus is the complexity of the chemical industry whose outputs can end up in a wide range of products and sectors. Furthermore, the data availability is very limited for the chemical sector, especially when compared to other sectors [31]. This is particularly true for downstream flows of the sector. Concentrating on the upstream flows of the chemical sector is sufficient when focusing on the supply side, e.g., feedstock choices and their impact on climate change. However, for investigating the impact of climate change mitigation options towards the use and end-of-life (e.g. material efficiency, waste treatment options), it is key to better integrate the downstream flows of the chemical sector [31]. This is particularly important for the chemical sector, since large parts of its carbon inputs are not directly emitted but sequestered in products. Only by covering the entire life-cycle can we get a proper grasp of the entire emissions of the chemical sector and their timing.

By linking the upstream chemical production of intermediates with the downstream production of plastics, their use in different sectors, and their end of life, the model could assess mitigation strategies throughout the whole product life. In that way also the impacts of the circular economy on mitigating climate change can be analyzed.

### Steps to enhance and further develop the NEDE model

**Fig. 2:**
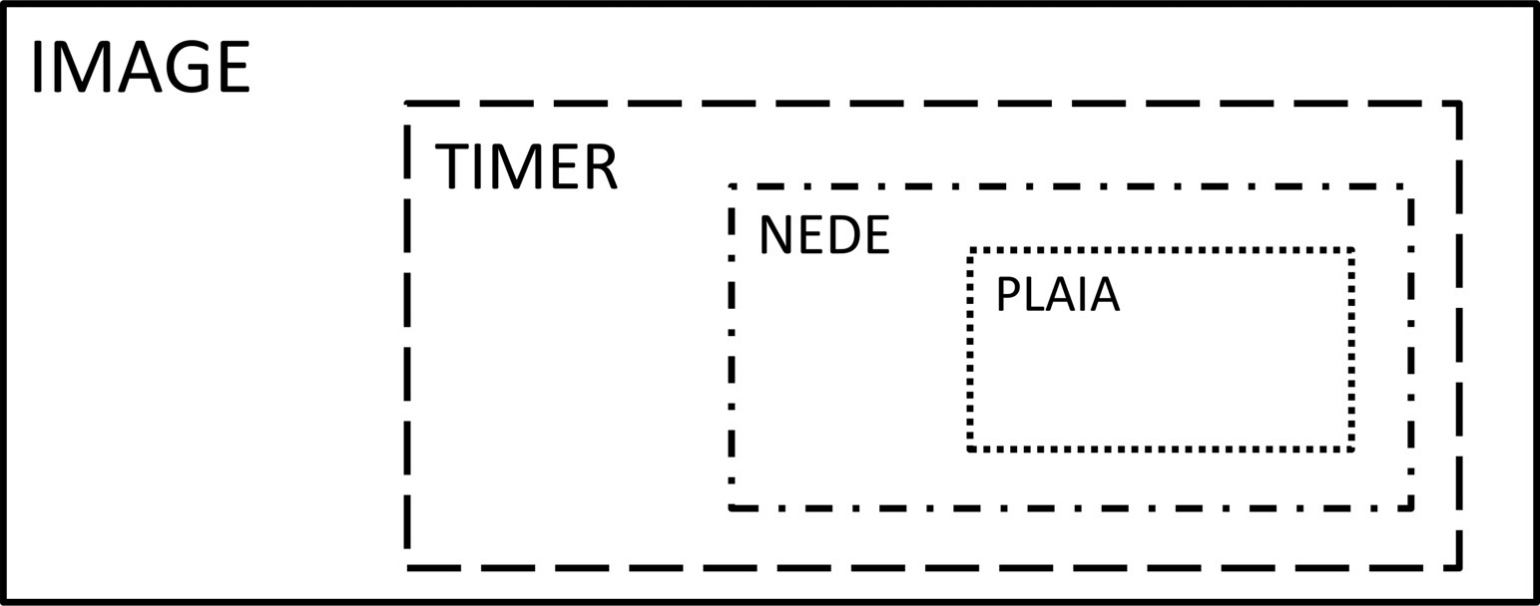
Structure within the IMAGE model: The IAM IMAGE incorporates the energy model TIMER. TIMER contains the sub-model NEDE, which covers the chemical sector. PLAIA is integrated in NEDE and models plastic production and waste management.

To that end, we created the plastics integrated assessment model (PLAIA) as an extension of the NEDE model. PLAIA added the representation of plastics, waste generation & stocks, and end-of-life options to the NEDE model and updated the model's Greenhouse-gas accounting accordingly. Fig. 2 shows the positions of the sub-modules referred to in this paper within the IAM IMAGE.

We created regional demand curves for chemicals from steam cracking by analyzing the relationship between GDP/capita and historic steam cracker capacities and by applying average product yields per steam cracker feedstocks. We defined the demand for plastics as a share of the demand for steam cracking products (i.e., ethylene, propylene, C4), refinery products (aromatics, propylene, C4 stream), and methanol. NEDE already provided us with the primary energy use of the upstream monomer production, to which we added the energy use for producing plastic polymers from these monomers and the transformation of polymers to plastic products. Furthermore, we modeled the plastic stocks in use and the yearly waste generation of plastics by defining the lifetime distribution of plastics by sector. We determined regional waste collection rates based on GDP/capita development. We added waste treatment technologies and modeled their shares in waste treatment based on costs and policy interventions. We also altered the existing carbon accounting system in NEDE to cover the implications of the end-of-life scenarios and carbon sequestration in products. This methodology paper is structured along with these steps. In Figure 15 in the SI, we show the structure of the updated NEDE model, indicating the additions of the PLAIA model.

### Overview of the Plastics Integrated Assessment model (PLAIA)

**Fig. 3:**
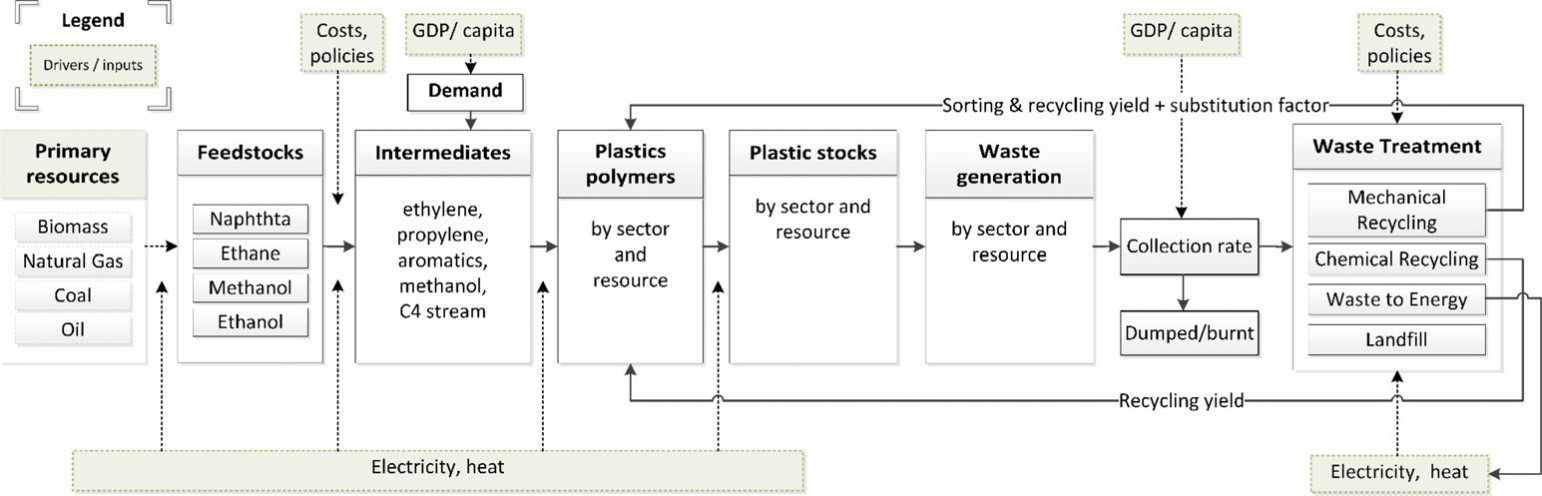
Overview of the plastics integrated assessment model (PLAIA).

PLAIA is integrated into the NEDE model and could be considered an add-on to the original NEDE model of Daioglou et al. [7]. Fig. 3 shows the structure of PLAIA. The model covers the plastics value chain from the extraction/production of primary resources (fossil & biomass) for the production of chemical feedstocks and intermediates to plastic polymer production, the transformation of polymers to products, their use in different sectors, and their end of life (= cradle to grave/cradle assessment).

The modeling steps and data sources behind PLAIA are described in detail in the following chapters.

## Creating demand curves for chemicals from steam cracking

The chemical sector is highly complex and has numerous and diverse technology routes and outputs. The data availability for the chemical sector is very limited, especially when compared to other industry sectors like steel and aluminum [31]. This makes it difficult to disaggregate the data used in NEDE to achieve a better representation of the outputs relevant to recycling, such as plastics.

The demand projections for high value chemicals (= steam cracking outputs) are based on data of the Oil & Gas Journal [35], which produces annual international surveys of Ethylene from Steam Crackers by country. This dataset provides the production capacity of global steam crackers in terms of feedstock (Ethane, propane, Butane, Naphtha, Gas Oil, Other) and ethylene output from 1980 to 2010. In its version of 2014 [7], NEDE only considered ethylene as an output from steam crackers. We complement this representation by also accounting for other steam cracker outputs like propylene, aromatics, and C4 streams. A relevant part of these streams is used for plastics production. We need to consider the full extent of steam cracker outputs to integrate plastics into NEDE successfully.

Using average steam cracking yields from Levi and Cullen [31] and the dataset mentioned above of the Oil & Gas Journal [35], we estimated the historic production capacity of ethylene, propylene, aromatics, and C4 streams for each IMAGE region. Using the lower heating values (LHVs) of Table 2 in the supplementary materials, we translated the mass-based yield matrix of Levi and Cullen [31] into energy-terms (GJ product/GJ feed) (see Table 3 in the supplementary materials). The resulting historic production capacity of HVC for all 26 IMAGE regions was then analyzed to determine the relationship between per capita production capacity growth and GDP per capita growth.

In the absence of actual demand data, we assumed historical production capacity data to represent demand. We model the HVC demand in NEDE as a logistic growth relationship between HVC production capacity per capita and GDP per capita (see Eq. 1), assuming a steam cracker utilization rate of 90%. Eq. 1 was selected as it proved to best match historical developments and expected behavior (i.e., demand growth levels off with higher GDP per capita).

**Eq. 1: Model for projecting HVC demand (in GJ) as a function of GDP/capita for an IMAGE Region R**

$$HVC_{R}=\;\alpha_{R}\;\times \;e^{-\frac{\beta_{R}}{GDP_{R}}}\;\times \;Pop_{R}$$

The coefficients Alpha and Beta were determined with regressions of the regional historical data. The regional historical data from the Oil & Gas Journal [35] is displayed in the SI (Figure 14).

Table 4 in the supplementary materials shows the final regression coefficients Alpha and Beta chosen as input for Eq. 1. We decided to limit Alpha to 13 to prevent the model from growing to unrealistically high future per capita consumption of HVC with very high economic growth (i.e., historical per capita consumption in most developed regions levels off below this value). Furthermore, choosing this limit of 13 allows for improved replication of historical production.

For the IMAGE regions USA, Western and Eastern Europe, Korea, South East Asia, and Japan, specific regression coefficients could be identified that fit the available historical data for those regions. Together, these regions covered 60% of global HVC production in 2010. For the remaining regions, the regression coefficients are based on all worldwide data points (excluding years with 0 HVC values). This is because most of the remaining regions still have limited historical data available or extremely low HVC production levels (and GDP per capita), making it nearly impossible to formulate an appropriate regression. We made exceptions for China and the Middle East, both significant producers of steam cracking products. As the data of China only reflects the early development of HVC capacity, no sound Alpha could be calculated. In the absence of better data, we fixed the Alpha for China at the value of the rest of the world (value of all global data points, see Table 4 in the supplementary materials) and calculated the Beta according to this Alpha and the existing data for China. For the Middle East, we based the regression coefficients on the global GDP/capita development instead of regional GDP/capita values. Our analysis showed that the production capacity in the Middle East does not have a strong correlation with its regional GDP development but rather the global one. This could be explained by the fact that its oil & gas industry is in large parts built for export.

## Integrating plastics demand into the model

### Overview

The chemical sector can be structured in upstream production and downstream production. The upstream production produces the primary or intermediate chemicals such as light olefins (ethylene, propylene), BTX aromatics (benzene, toluene, xylene), ammonia, methanol, and a C4-stream (e.g., Butadiene, Isobutene). The production of these intermediates is responsible for around two-thirds of the energy consumption in the chemical sector (feedstock and process energy combined). The downstream production includes plastic polymers, agrochemicals (e.g., fertilizers, surfactants, pesticides), and specialty chemicals (e.g., solvents, paints, industry catalysts). These products are then further processed for final use in, e.g., packaging, agriculture, construction, or pharmaceuticals (International Energy Agency (IEA) [26,31]).

**Fig. 4:**
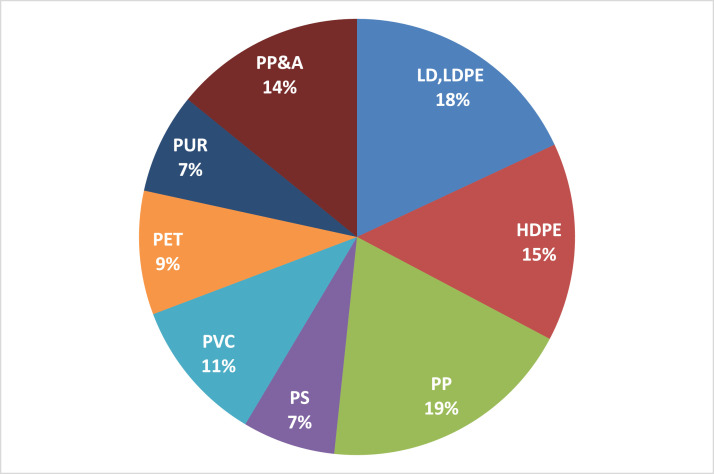
Shares of plastic types in the global market (Adapted from [18]).

Fig. 4 shows the major plastic types on the market, which are relatively constant over time (2002 – 2014) and analyzed regions (EU, USA, China, India) according to Geyer et al. [18]. In plastic resin production, the upstream production of monomers like ethylene and propylene is responsible for most energy use and emissions. For example, for PP, LDPE, LLDPE and HDPE, the energy use of the upstream monomer production is around 90% of total energy use and is responsible for about 80% of the global warming potential of plastic resin production [39,40]. After the upstream monomer production, some monomers are processed to further intermediates like ethylene glycol, styrene, terephthalic acid, or vinyl chloride before the final polymerization step. During polymerization, monomers are chemically bound in chains to plastic polymers [1,31] and then converted and manufactured into their final products.

The NEDE model covers the upstream chemical production from steam cracking, refinery, ammonia, and methanol production as described in Daioglou et al. [7]. To integrate plastics into the model, we analyzed the shares of the upstream chemical intermediate products going to plastics. Then, we added the downstream production steps to the model (polymerization and transformation).

### Determining the energy content of plastics

TIMER is an energy model, and as such, it deals in energy units. Therefore we integrated plastics not in mass but energy terms. For that purpose, we define an average lower heating value (LHV) of plastics to convert the data from mass into energy units and vice versa.

We calculated average LHVs for each plastic type based on values collected from the literature (see Table 5 in the supplementary materials). We multiplied them with the global shares of these plastic types on the market (from [18], see Fig. 4). This provided us with a weighted average LHV of 35 GJ/t for plastics, which is within ranges provided in literature as a plastic average [55]. Using this LHV, we converted the plastic data from mass to energy units and vice versa. We also used this LHV for calculating the benefits of incinerating plastic waste in waste to energy plants. We assume this LHV to stay constant over time. This assumption is discussed further in the final section of this article on model limitations.

### Defining the upstream sources of plastic production

NEDE structures the demand for non-energy/chemical use into the aggregated categories high value chemicals (olefins coming from steam cracking), refinery products (aromatics, bitumen, lubricants), methanol, and ammonia. Most intermediate feedstocks for plastic production (see chapter 3.1) originate from steam cracking, but also olefins and aromatics are sourced in significant numbers from refineries (163 million tonnes in 2013, according to [31]). Furthermore, plastic feedstocks also originate from methanol: the methanol-to-olefins (MTO) production is growing, and also other methanol-sourced products like acetic acid or formaldehyde end up in plastics [33].

Unfortunately, there is very little information on the flows within the chemical sector. Levi and Cullen [31] provide an overview of the global flows within the chemical sector for the year 2013 based on the limited available information. In the absence of other comprehensive data sources and historical material flows in the chemical sector, we used their data to define the shares of intermediate chemicals from steam cracking, refinery sourced olefins, and aromatics, and methanol going to plastics.

Using the data of Levi and Cullen [31], we defined the shares of ethylene, propylene, BTX aromatics, methanol, and C4 (upstream chemical production) going to plastics. Then we allocated these streams to the NEDE categories HVC, methanol, and refinery products, based on the share of these categories in the respective upstream intermediates. In a third step, we defined the LHVs of these intermediates and converted the mass units of Levi and Cullen [31] into energy units to adopt them to the NEDE model (million metric tonnes to Petajoule). We used those converted values to calculate the shares of steam cracking products (0,84), refinery sourced olefins & aromatics (0,59), and methanol (0,4) going to plastics (in energy terms). In the absence of historical data, we assume these shares to remain constant throughout the years.

### Adding refinery-sourced olefins to the model

In the model version of Daioglou et al. [7], refinery products are based on historic production capacity data by country from the yearly "Worldwide Refining Survey" [36] and cover bitumen, lubricants, and aromatics. However, this data does not include olefins originating from refineries such as the C4-stream and propylene (via fluid catalytic cracking, FCC) [31,40]. Their full consideration in the model is important, as 32% of the monomers used in plastic production come from refinery-sourced aromatics, propylene, and C4 [31]. In the absence of better data, we adopt a simplified representation of refinery-sourced propylene and C4 as a function of aromatics produced in refineries. The refinery-sourced aromatics are modeled based on historical data from the Oil & Gas Journal (see [7]). We express the demand for refinery sourced propylene and C4 as a fixed factor of 0.5 GJ propylene and 0.8 GJ C4 demanded per GJ of refinery sourced aromatics. These relationships between propylene and C4 to refinery-sourced aromatics are based on data of Levi and Cullen [31].

### Determining the demand for plastics

Geyer et al. [18] provide historical data for global primary plastic production. However, regional historical production or demand data is scarce. There is (partial) plastic production data publicly available for some world regions or countries, but not consistent, and it does not cover all relevant regions. A regional representation of the chemicals and plastics sector is essential for the IMAGE model, as it provides results for 26 world regions.

The data used in NEDE for modeling the demand for chemical intermediates (see [7]) offers more granularity: The Oil & Gas Journal [[35], [36]] provides country-specific data for refineries and steam crackers and the Methanol Institute [33] for methanol production. Therefore, we chose to represent plastic demand as a function of the upstream chemical production data. This approach allows for a regional disaggregation of plastic demand in the model.

Using the shares of upstream chemical products going to plastics, we defined plastic demand (P) as a function of the demand for the NEDE product categories high value chemicals (HVC), refinery products (RP) and methanol (M) over time (t).

**Eq. (2): Plastic demand as a function of demand for upstream chemical products over time**

P(t)=0.84*HVC(t)+0.3*RP(t)+0.4*M(t)

In the final section of this article (Discussion of limitations), we compare the results of our approach with other global projections of plastic demand.

## Primary plastic production

### Upstream monomer production

The upstream chemical production of monomers covers most of the energy use and emissions in plastic production. These monomers and chemical intermediates used in plastic production (i.e., ethylene, propylene, aromatics, C4 stream, methanol) come in large parts from steam crackers but are also sourced from refineries and complemented by methanol. In the model, these chemical intermediates can be produced via various technology routes using coal, oil, natural gas, and biomass as feedstock. This upstream chemical production, its costs, and energy use are represented in the model as described in Daioglou et al. [7].

### Polymer production

Plastic monomers are processed into plastic polymer resins. We calculated the weighted average electricity and heat use of plastic resin production using production data for HDPE, LDPE, LLDPE, PP, PET, PVC, and PS from PlasticsEurope [39], [40], [41], [42] multiplied by their respective market share (see Fig. 4). Together, these plastic polymers cover almost 80% of the plastics market [18]. Therefore, we chose them as a proxy for the entire market. The energy use covers the final polymerization step and intermediate steps like the production of ethylene glycol, purified terephthalic acid, chlorine, and vinyl chloride production. Table 6 in the supplementary materials shows the electricity and heat use.

Monomer production is the most significant cost driver of plastic production compared to the downstream production steps, such as polymerization: Looking at global price differences between olefin monomers (i.e., ethylene, propylene) and polymers (i.e., LDPE, HDPE, PP), we usually see that the olefin price fluctuates around 80% of the polymer price^3^. Most reports only refer to a total plastic resin costs or price and do not detail the share of monomer production and polymer production, see e.g., Hestin et al. [23] or Villanueva and Eder [55].

We take the costs of the process heat and electricity requirement as a proxy for the costs of producing plastic polymer resins from monomers. The costs of electricity and heat are endogenously modeled in IMAGE TIMER and are region-specific and dynamically modeled over time, see PBL [37] and Stehfest et al. [53]. While this does not cover the entire costs of the polymerization process, it is a reasonable proxy. Energy use is the most significant cost factor for plastic resin producers, typically around 70% [54].

### Polymer transformation into products

Plastic resins are further transformed into semi-finished plastic products via different technologies like calendaring, blow molding, compression molding, extrusion, or injection molding [29]. As it is difficult to capture the entire variety of plastic products in a long-term global-scale model, we chose to include a proxy value for the energy use of plastic transformation.

We calculated a weighted average energy use and efficiency of plastic transformation per plastic type, based on data by Keoleian et al. [29], who collected energy use and efficiency data for nine transformation technologies and data on the share of these technologies for transforming different plastic types. Combining this data of Keoleian et al [29]. with the market shares of the plastic polymer types of Geyer et al. [18], we calculated an overall weighted average energy use and efficiency for plastics transformation (see Table 7 in the supplementary materials, translated into the metric system).

The data of Keoleian et al. [29] did not cover all plastic types but included PP, PVC, PET, and PE (data applied for both LDPE/LLDPE & HDPE). Together, they cover 72% of the plastic market, according to Geyer et al. [18], and were thus chosen as a proxy for the entire plastic market. For simplification purposes, we assume that the entire energy use comes from electricity which is mainly in line with Keoleian et al. [29] (apart from blow molding) and Franklin Associates [16], who state that 97% of energy use in injection molding is from electricity and 95% in thermoforming.

As for the cost of polymerization, we just account for energy use costs, using the electricity prices generated in TIMER. We also apply the plastic transformation to recycled plastic resins.

## Plastic stocks & plastic waste generation

**Fig. 5:**
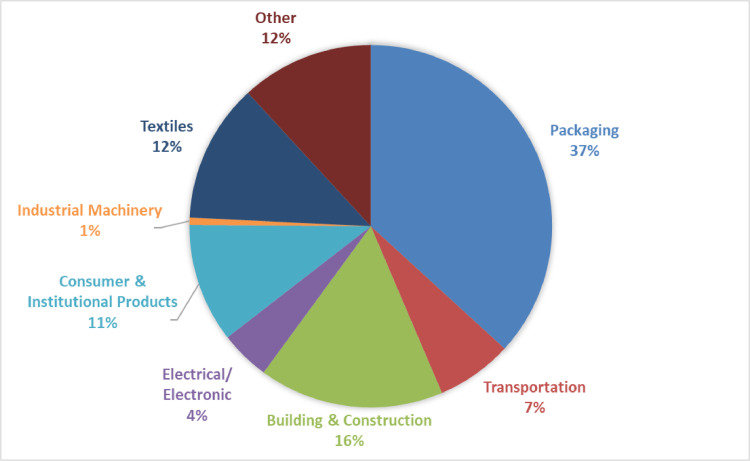
Sector shares in plastic demand; own illustration based on data compiled by Geyer et al. [18]; data for 2002-2014 from Europe, the United States, China, and India.

Plastics are used in various products and sectors. The model distinguishes between eight sectors of which packaging is the biggest in yearly production with around 37% (see Fig. 5).

**Fig. 6:**
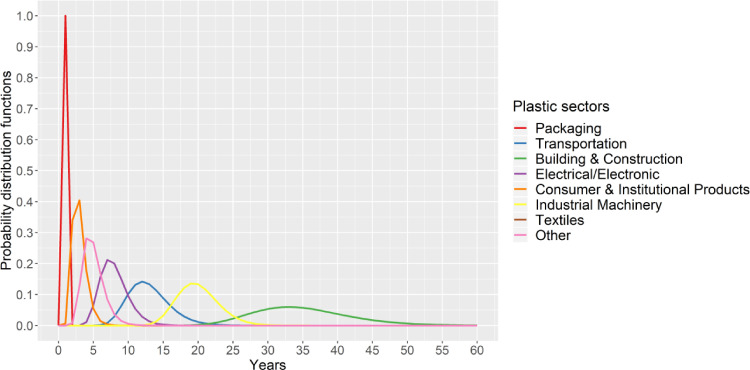
Plastic product lifetime distributions; Own illustration based on data compiled by Geyer, Jambeck, and Law [18].

The duration of the use phase of plastics varies significantly between the sectors. While plastics in packaging usually become waste within a very short time (typically less than a year), plastics used in building and construction have a much longer time of use and thus often become waste decades later. We defined sector-specific lifetimes of plastic products via lognormal probability distribution functions based on data compiled by Geyer et al [18], see Fig. 6. Geyer et al [18] assumed that the sector "Other" has the same distribution as textiles.

Table 8 in the supplementary materials shows the mean use time of plastics and their standard deviation per sector as compiled by Geyer et al. [18].

Based on the use time of plastic products, we calculate the plastic waste (PW) generated each year (t) per region (R) and sector (S) as the sum of the plastics (P) produced in year (t-n) becoming waste in year t. n is the number of years to look back for plastic production data, with an upper limit of 60 years (maximum lifetime of plastics in building & construction, see Fig. 6). The distribution factor (d) defines the share of plastics produced in year t-n becoming waste each year, according to the probability distribution in Fig. 6.

**Eq. (3): Calculating the annual plastic waste generation**

$$PW_{R,S}\left(t\right)=\sum_{t=1}^{n}d_{S}\left(t\right)\;\times \;P_{R,S}\left(t-n\right)$$

Having the yearly plastic production and waste generation, we can calculate the plastics stocks (= plastics in use) in each of the eight use sectors. We define the Plastic Stock (PS) per sector (S) and region (R) in year t as the cumulative plastics produced (cP) minus the cumulative plastic waste generated (cPW):

**Eq. (4): Calculating the plastic stocks (= plastics in use)**

$$PS_{R,S}\left(t\right)\;=cP_{R,S}\left(t\right)\;-\;cPW_{R,S}\left(t\right)$$

## Plastic waste collection

Collection rates describe the share of generated waste that is collected and thus entering the waste management system. In the model, the collected waste is then available for the different waste treatment options like landfilling, energy recovery, mechanical and chemical recycling. The waste that is not collected is assumed to be openly burnt or dumped, potentially ending up in waterways and oceans.

We model the waste collected by region as a function of GDP per capita, using data collected by the World Bank [51]. The data of the World Bank provides collection rates for four levels of Gross National Income (GNI) ranges per capita, see Table 9 in the supplementary materials. We assume that the GNI/cap equals GDP/capita to allow for an integration of these numbers into the model. Differences between GNI and GDP are usually marginal, i.e., ranging from 0.1-2%. We smoothened the transition between income levels to avoid sudden jumps in collection rates once a region passes the World Bank thresholds.

## Plastic waste treatment

### Overview

In the model, the amount of collected plastic waste can be directed to mechanical recycling, chemical recycling (via pyrolysis), waste to energy, or landfilling. The collected plastic waste is allocated to the different plastic waste treatment options (WTO) based on the WTO's relative costs, policy interventions (e.g., CO_2_ tax, bans), and technological or economic constraints (e.g., maximum recyclability).

The costs of the waste treatment options consist of a fixed cost factor, endogenously modeled variable costs (for heat, electricity, and diesel use), and a CO_2_ tax (if part of the modeled scenarios). These costs are reduced by the endogenously modeled benefits of replacing primary plastics (for mechanical & chemical recycling) or heat and electricity (for waste to energy). The costs exclude the collection & transportation of plastic waste. Table 10 in the supplementary materials summarizes the data used for modeling the waste treatment options in PLAIA. The data and assumptions for each option and calculation steps are explained in this chapter.

### Data & assumptions for the waste treatment options

#### Mechanical recycling

The process of mechanical recycling can be structured in a simplified manner in (1) collection, (2) sorting/pre-treatment, (3) transportation to a recycling plant (and transportation of sorting and recycling rejects to Energy recovery or landfilling), and (4) recycling.

##### Collection & transportation

The energy use and emissions of the collection and transportation steps are related to transport distances for refuse collection and distances to waste treatment options. These steps are excluded from the model as it is challenging to find representative data for all IMAGE regions on average transportation distances. Furthermore, the literature showed that these steps have a comparably small impact on the recycling process's overall energy use and GHG emissions [23,60]. We discuss this further in the final section on limitations.

##### Sorting & recycling yields

Sorting/pre-treatment yields for plastics vary by sector. We took a weighted average of 0.75 based on data from Hestin et al. [23], who provide information on sector-specific sorting yields for Europe.

The term recycling rate is used differently through literature and reporting schemes, leading to overestimating recycling. It often refers to the number of plastics sent to recycling (i.e., the input recycling rate). It thus ignores all the process losses along the way in sorting and recycling (output recycling rate).

We use the term recycling yield to describe the process efficiency of recycling itself (excl. collection rate and sorting yields), so the amount of plastic resin produced per sorted and pre-treated waste input. Many sources describe yields for specific plastic resins and technologies which achieve relatively high rates for some resins (e.g. 0.88 for PET, see [14]), while other plastic resins show much lower yields. We chose a weighted average recycling yield of 0.75 for the model, based on the European industry data compiled by Hestin et al. [23], covering the most relevant plastic resins. This value is also within the range of other sources, estimating the overall plastic recycling yield to be within 0.7 and 0.78 [61] or 0.7 – 0.82 (International Energy Agency (IEA) [26]).

We define the overall recycling efficiency (RE) as the product of the sorting (SY) and recycling yield (RY):

**Eq. (5): Recycling efficiency**

RE=SY*RY

Eq. 6 leads to an overall recycling efficiency of 0.56.

##### Treatment of rejects

A significant part of plastic waste sent to mechanical recycling will not end up in recycled plastics due to the losses in sorting and recycling processes. In the model, these rejects of mechanical recycling are sent to the other waste treatment options according to their market shares in plastic waste treatment.

##### Energy use

The model covers the heat and electricity use of the sorting/pre-treatment and the recycling step. We identified in the literature that values for electricity use in sorting plastic waste range from 20 to 105 kWh/t plastic waste [2,14,22,23,45,59]. We chose the average of the electricity consumption for sorting reported in the literature as input into the model (see Table 10 in supplementary materials).

We took data on electricity and heat requirements for recycling plastic waste from Faraca et al. [14]. Faraca et al. [14] provide electricity and heat use data for recycling different polymers (PP, PE, PET, PS) based on a review of 11 studies. By multiplying these energy requirements with the polymer market shares of Geyer et al. [18], see Fig. 4, we calculated the weighted average heat and electricity use for mechanical plastic waste recycling (see Table 10 in the supplementary materials). This average does not consider all polymers, but it represents almost 70% of the plastics market.

##### Costs

The costs of mechanical recycling in the model cover the sorting/pre-treatment and recycling of plastics. They consist of (1) a fixed cost factor (incl. machinery and labour), (2) electricity and (3) heat costs, and (4) a CO_2_ price based on the emissions of the process (for those scenarios with CO_2_ price).

For the fixed cost factor, we took data from a study of Plastics Recyclers Europe [23], which provides costs data per plastic sector and an average. The data of Hestin et al. [23] is collected from European recycling plants and is in the range of other cost data reported in the literature [14,21].

Costs for electricity and heat are around 10% of total mechanical recycling costs in Denmark and the United Kingdom [14,62]. We deducted 10% of the fixed cost factor from Hestin et al. [23] and added endogenously calculated variable costs for heat and electricity from the TIMER model. We display the cost data in Table 10 in the supplementary materials.

##### Substitution rate

In how far recycled plastics can replace primary plastics depends on their quality. This quality is usually lower, which is also reflected in the price of recycled plastics [12,61]. This difference can be expressed by a substitution factor/rate. We set this substitution rate to 0.81 in the model, based on European Commission [12] and Rigamonti et al. [45]. The use of this substitution rate in the model captures the price differences between primary and recycled plastics. The price recycled plastics achieve on the market lowers the recycling costs and thus influences the share of recycling in the model (see Eq. (7)).

##### Cost of primary plastics production as a cost driver for recycling

The cost of primary plastics is a strong driver of recycling rates. If it is high, the demand for secondary plastics rises; if it is low (e.g., due to a low oil price), there is little incentive for recycling. To cover this effect in the model, we define the total costs of mechanical recycling (MRcosts) for Region R in year t as follows:

**Eq. (6): Calculating the overall cost of mechanical recycling**

$$MRcost_{R}\left(t\right)\;=\;\left(FCF_{R}\left(t\right)+Celec_{R}\left(t\right)\;+\;Cheat_{R}\;\left(t\right)\right)\;-\;SF\;\left(t\right)\;*\;CPP_{R(}\left(t\right)$$

With FCF being the fixed cost factor, Celec being the costs of electricity, Cheat the costs of heat, SF the substitution factor, and CPP the costs of primary plastics production.

#### Chemical recycling via pyrolysis

The model includes a chemical recycling route via pyrolysis. The route is a three-step process that (1) transforms plastic waste into naphtha, (2) naphtha into chemical intermediates via steam-cracking (e.g., ethylene), and (3) into plastic polymers via polymerization.

This process also has a preceding sorting step. We took over the same electricity values as for sorting for mechanical recycling. However, we used a higher sorting yield of 0.85 [14], as pyrolysis tolerates more impurities and mixed-plastics than mechanical recycling. Only some plastic types like PVC could cause processing problems and have to be sorted out.

Pyrolysis was already part of the original NEDE model. Therefore, the technology data (costs, efficiency, and energy-use) are described in Daioglou et al [7]. We only added the pre-sorting step and the polymer production process as described in the respective section. Table 10 in the supplementary materials summarizes the yield and costs of the production of naphtha from plastic waste and the transformation of naphtha to monomers.

Plastics produced via chemical recycling can achieve the same quality as primary plastics. Therefore a substitution factor of 1 is assumed (unlike for mechanically recycled plastics, see the previous section). We deduct the financial benefit of replacing primary plastics with chemically recycled plastics from the chemical recycling costs (same as for mechanical recycling, see Eq. 7). As chemical recycling is a hi-tech category mainly applied in high-income economies, we activate this technology only for regions that surpass a GDP/cap of 10,000 USD. Historically, chemically recycling played a notable role only in Japan. Therefore, chemical recycling is not considered in the historical model results (before 2021) apart from Japan.

#### Waste to energy

Waste incineration technology is traditionally mainly used in high-income countries like Japan, the USA, and many European countries [28,47,51]. In recent years, also China drastically increased its waste to energy capacity [47]. High land prices, high technical capacity, and high financial resources favor waste to energy [28]. Furthermore, a waste composition with a high calorific value is important, which means that the share of organic waste should be low (which is usually not the case in low-income economies). To reflect this in the model, we allow waste to energy as an option only for regions that surpass a GDP/capita of 10,000 USD/capita (2005 USD).

##### Heat & electricity generation efficiency

Modern incineration plants are with energy recovery and can be optimized for heat or electricity generation, resulting in a wide range of potential efficiencies. In Europe, 59 million tonnes of waste is treated in 314 waste to energy plants with an average heat and electricity efficiency of 34.6% and 14.9%, respectively [12]. At the same time, almost 36 million tonnes of waste are incinerated without energy recovery in Europe. Using the weighted average of the European Commission [12], we assume an average heat efficiency of 22% and an electricity efficiency of 9% in the model, representing a total waste incineration capacity of 94.7 million tonnes with and without energy recovery.

We assume the same technology data globally in the current model version. While we expect increases in average generation yields for Europe in the future, emerging economies might struggle with imperfect waste-to-energy processes in the initial years. Therefore, this weighted average seems more suitable for defining a global value for generation efficiencies.

##### Costs

In the model, we assume fixed total costs of plastic waste incineration and deduct the variable revenue from selling the generated heat and electricity. Additionally, we apply a CO_2_ price on the fossil share of incinerated plastic waste (in the scenarios where a CO_2_ price is present). The incineration of bio-based plastic is exempt from this CO_2_ price. The costs and energy generation efficiency are shown in Table 10 in the supplementary materials.

The costs of plastic waste incineration used in the model are from a Dutch Waste-to-energy plant and include operational and capital expenditure and bottom ash treatment [21]. The costs of this plant are similar to other values reported in the literature, e.g., of a Danish waste to energy plant [14] and a German plant [24]. Furthermore, it is within the range of gate fees for incinerators in the European Union [55]. However, these gate fees also include taxes and the credit from selling energy [55].

The variable benefit of selling the generated electricity and heat is based on the generation efficiencies and the regional heat and electricity price generated in TIMER.

#### Landfilling

Landfilling is globally the dominant end-of-life option, but the quality of landfilling and the costs can differ significantly from open dumps, controlled landfills to properly engineered sanitary landfills [28,51].

##### Energy use

Energy use plays a small role in operating landfills compared to the other end-of-life options. Per tonne of waste, 1.5-4 l of diesel are used for excavation works and daily operations and 5 to 8 kWh electricity [32]. We used an average of these values for PLAIA; see Table 10 in the supplementary materials.

##### Costs

Landfill costs strongly vary between locations, ranging from a minimum of 10 USD/t in low-income countries to 100 USD/t in high-income countries (Silpa [51]). Including landfill taxes, the costs could increase further: for example, Sweden has an average landfill cost of 155 Euro/t, and individual landfills could also be more costly as an example of 219 Euro/t in Austria shows (European Environmental Agency (EEA) [13]).

The costs depend on *"terrain, soil type, climatic factors, site restrictions and regulatory factors,"* as well as the type of waste and the volume of waste received [10]. Capital costs (incl. site development, closure & post-closure) have the highest share in total costs. Operational and Monitoring costs also vary: They account for 38% of total costs in an example of the United Kingdom [24] and 44% in an example of rural Oklahoma in the USA [10].

In the model, we differentiate between three cost factors:

1. Fixed costs, including Site Development & Construction, Equipment, personnel, monitoring, closure & post-closure, taxes
2. Electricity costs for operation
3. Diesel use for excavation works & daily on-site operations.

The use of electricity and diesel is based on Manfredi et al. [32], see energy use above, and the costs are calculated via the respective energy prices generated in TIMER. They only account for a very marginal share in total landfill costs in the model, ranging from mostly less than 1% up to ca. 5% for some regions.

The dominant cost factor is the fixed costs which can vary significantly as described above. These differences are not just due to location-specific conditions (e.g. terrain, climatic factors, scale, personnel costs) but are also driven by regulations, policies, and suitable land availability. This leads to major differences also between high-income countries: While less densely populated countries like the USA or Australia show a high share of landfilling and lower costs, Japan and countries within the European Union have higher costs and lower shares going to landfills [5,13,38,57].

Covering these dynamics in PLAIA is a challenge. We chose to model regional and future variations in costs of landfilling based on differences in land prices, representing the GDP & population density of a region. The assumption behind our approach is that with rising GDP & population density also costs of landfilling rise, and regions increasingly switch to recycling and waste to energy. We calibrated the model by defining a baseline value Alpha for landfilling costs that keeps the model within realistic landfill cost ranges as reported in the literature [5,13,51,57]. PLAIA uses the dynamic land prices generated in IMAGE, which are based on GDP per capita, population size, and usable area in each IMAGE region. We calculate the regional costs of landfilling in PLAIA as follows:

**Eq. 7: Costs of landfilling in 2005 USD/GJ plastic waste**

$$\text{Landfill}\;\text{Cost}s_{R}\left(t\right)=\;\frac{LP_{R}\left(t\right)}{LPav_{Global}\left(t\right)}\;*\;\alpha \;*\;\frac{LPav_{Global}\left(t\right)}{\beta }\;+\;\;EC_{R,i}\left(t\right)$$

With $LP_{R}\left(t\right)$ being the land price in year t for region R, $LPav_{Global}\left(t\right)$ being the weighted global average land price (weighted based on region size) and $\;EC_{R,i}$ are the costs of electricity and diesel use for landfilling per region. Alpha represents the chosen baseline fixed costs for landfilling (set as the global average in 2015) and Beta the baseline global average land price in 2015 (generated in IMAGE). This means that all future landfill costs are calculated (a) in relation to the base year 2015 and (b) changes in regional land prices and (c) regional energy prices.

#### Open burning & dumping

In PLAIA, plastic waste collection is driven by GDP/cap development (see chapter 6). The difference between plastic waste generated and collected is assumed to be openly burned or dumped/littered in the environment. We assume that 30% of this remaining uncollected waste will be burned and 70% dumped. This estimation is based on World Bank data, which provides the share of openly burned or dumped waste for some countries [51]. However, this estimation is uncertain as it is difficult to cover these informal waste disposal methods in national statistics.

### Defining the market shares of the waste treatment options

The plastic waste entering the waste management system (= collected plastic waste) is allocated to the different plastic waste treatment options (WTO) based on (1) the WTO's relative costs (a described in chapter 7.2), (2) policy interventions (e.g., CO_2_ price, bans) and (3) technological or economic constraints (e.g., maximum recyclability).

For defining the shares of the WTOs we use a multinomial logit function as shown in Eq. 9, with C being the cost of each WTO and Region (R) and λ being the logit parameter which defines the elasticity between relative prices. This function allocates market shares amongst WTOs based on their relative costs. Thus, even non-economically optimal options get selected to small extents. Such a function allows for the representation of heterogeneity in waste management and takes into account that, in reality, decisions are not purely economic.

**Eq. 8: Multinomial logit function to calculate the market shares of waste treatment options**

$$WTO\_Share_{R,\;WTO}\;=\;\frac{e^{-\lambda \;*\;C_{R,\;WTO}}}{\sum_{WTO\;}e^{-\lambda \;*\;C_{R,\;WTO}}}$$

The hi-tech WTOs waste to energy and chemical recycling are only active in regions that surpass a GDP/capita of 10,000 USD (2005).

## Carbon accounting

PLAIA accounts for the carbon flows throughout the entire life cycle of plastics: from the primary energy carrier to the products, their use, and finally, their end-of-life. Also, transformation losses and carbon emissions from heat and electricity use in production and waste management are covered. The model differentiates between the total carbon input to the plastics sector, the total carbon emissions, total carbon in use (sequestered in products), total carbon sequestered in landfills/dumps, and the total carbon recycled. Furthermore, it specifies emissions in plastic production and end of life for the different waste treatment options.

The emission accounting is in line with the 'Good Practice' methods described by the IPCC guidelines for emission inventories [9]. Only fossil carbon is accounted for in emissions as bio-based carbon is assumed to be climate neutral. We include the agricultural process emissions and land-use change emissions of biomass production (calculated in IMAGE). Indirect land-use change emissions are not relevant in IMAGE as the model adopts a food-first principle [6]. Also the production emissions of fossil energy carriers are included.

According to the International Energy Agency (IEA) [26], emissions of the chemical sector can be distinguished in

1. Energy-related emissions: Emissions created by process energy use. They are responsible for the largest share in emissions, with around 85%.
2. Process emissions: Emission occurring when transforming the feedstock into the product. Those are expressed by the difference in the carbon content of the feedstock (e.g., methane, oil) and the carbon content of the product (e.g., Ammonia, Plastic) and are responsible for around 15% of the sector's emissions.

In PLAIA, all fossil energy carriers used for process energy are assumed to be directly emitted (= Energy-related emissions) unless carbon capture & storage technology is applied. The carbon of fossil energy carriers used as a feedstock for short-lived products (e.g., lubricants, solvents, ammonia, cosmetics, etc.) are assumed to be emitted in the same year of production. For fossil energy carriers used as feedstock in products with a lifetime of more than a year (e.g., plastics), the difference in carbon between the feedstocks and the products is assumed to be emitted, following the example of the International Energy Agency (IEA) [26].

The future fate of the carbon embedded in plastic products (i.e., recycled, landfilled, incinerated) is determined by the waste management practices in the year the lifetime of the plastic product ends. We assume that plastic carbon going to landfills and dumps stays sequestered for the entire analyzed period (up to 2100). We went for this simplified assumption as there is very little literature on the degradation of plastics in different environments [4] and even less on its implications on Greenhouse-gas emissions [48]. Moreover, plastic buried in landfills seems to have a very slow chemical degradation rate, as discussed in the final section on limitations [4,48]. For PLAIA, this means that within the model time frame until 2100, all carbon input to the plastics sector is ultimately emitted, apart from the carbon embedded in plastic products in use until 2100, and carbon stored in landfills or dumps.

We calculate the annual carbon balance as follows: Total carbon input (incl. waste generated) – Total carbon sequestered in products with a lifetime of >1 year – Total carbon additions to landfills and dumps. To calculate the annual emission balance in PLAIA, we need to define the carbon contents of plastics. We calculate the carbon content based on the shares of the feedstock energy carriers used for producing plastics, i.e., a weighted average of the carbon contents of coal, oil, natural gas, and biomass bound in plastics. Depending on the shares of these energy carriers in plastics, the carbon content of plastics differs per region and year. While this approach creates an inherently consistent carbon balance in the model, it also has disadvantages (see final section on limitations).

## Model outputs

The purpose of the PLAIA model is to assess the plastics sector's long-term material flows, energy use, and GHG emissions for different scenarios. The results of PLAIA for different scenarios are presented and discussed in other publications that are currently under review or in preparation. This section merely displays key outputs of the model for one baseline scenario to illustrate the types of results PLAIA can provide.

PLAIA was designed to run scenarios based on the shared socioeconomic pathways (SSP) and variations thereof. The climate change research community developed the SSPs as a set of alternative futures for societal development [34,44]. The illustrative results presented here are based on the second shared socioeconomic pathway (SSP2), which is closely linked to historical patterns in social, economic, and technological developments [17,34].

**Fig. 7:**
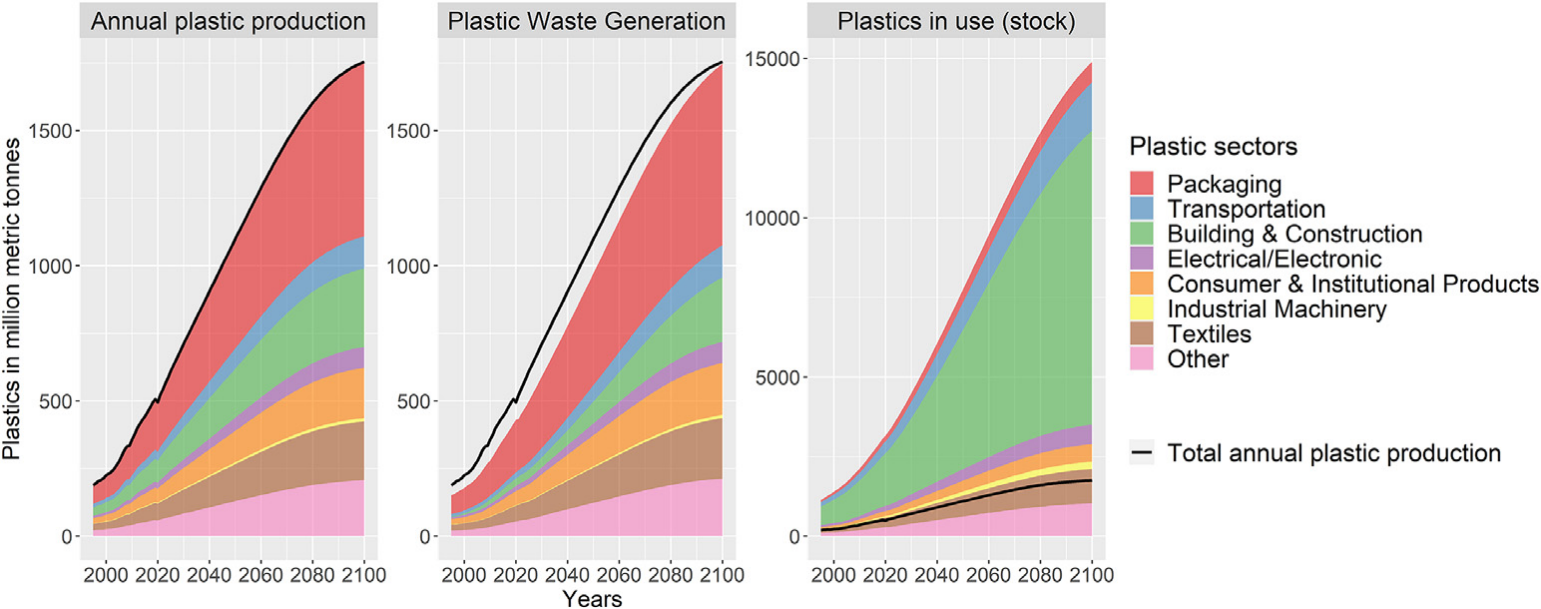
Projections of plastic production, waste generation, and stocks by sector (different scale for plastics stocks).

PLAIA projects the annual plastic production, annual waste generation and plastics stocks based on population and economic development as provided by the SSPs (see Fig. 7 for a SSP2 baseline).

**Fig. 8:**
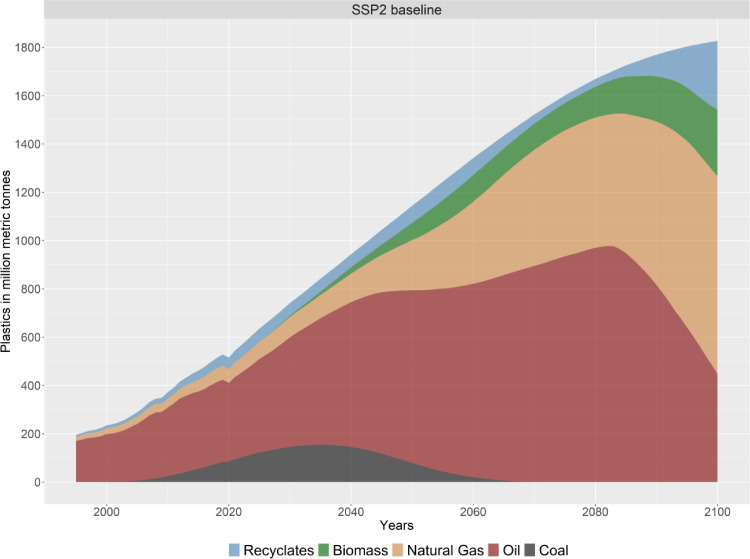
The global annual plastic production by feedstocks used.

Furthermore, PLAIA details the feedstocks used for plastics production (see Fig. 8).

**Fig. 9:**
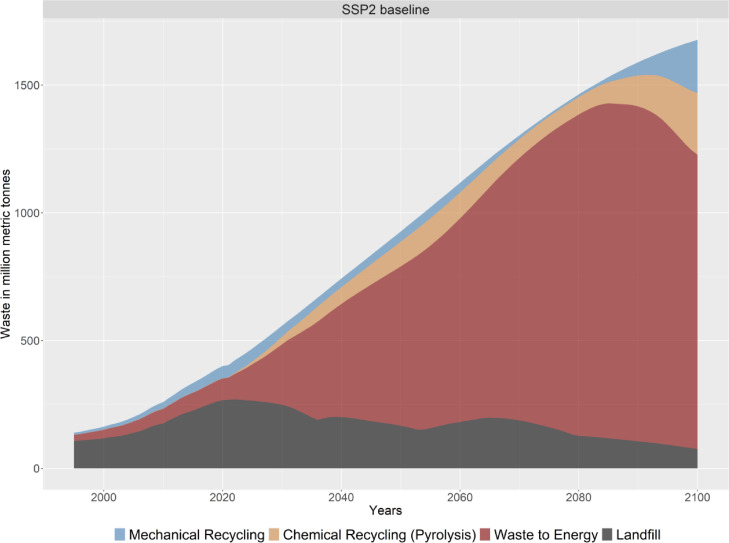
The global annual collected plastic waste and its fate (recycled, incinerated, landfilled).

Moreover, the model shows how much plastic waste is collected and how it is treated (see Fig. 9)

**Fig. 10:**
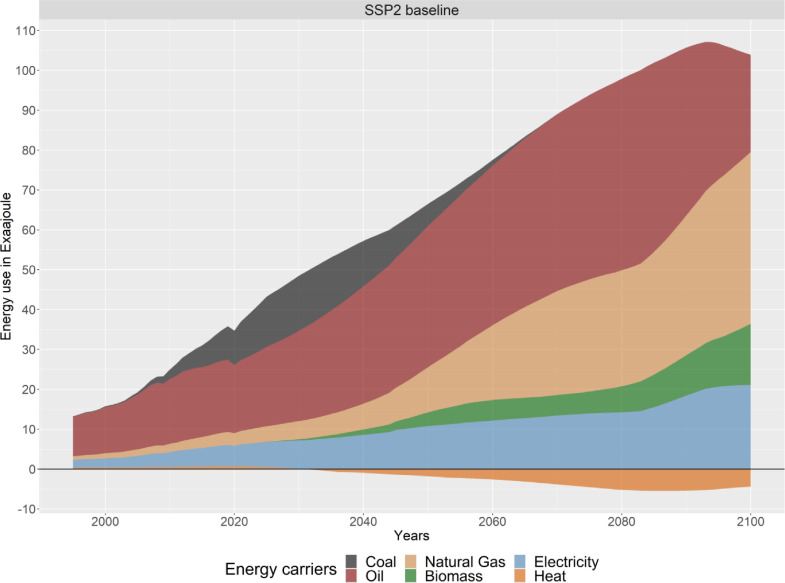
Global final energy use in the plastics sector over the entire life cycle (Heat use can become negative if more heat is produced via waste-to-energy than consumed by the sector).

PLAIA summarizes the final energy use of the plastics sector over the entire life cycle, detailing the types of energy carriers used (see Fig. 10).

**Fig. 11:**
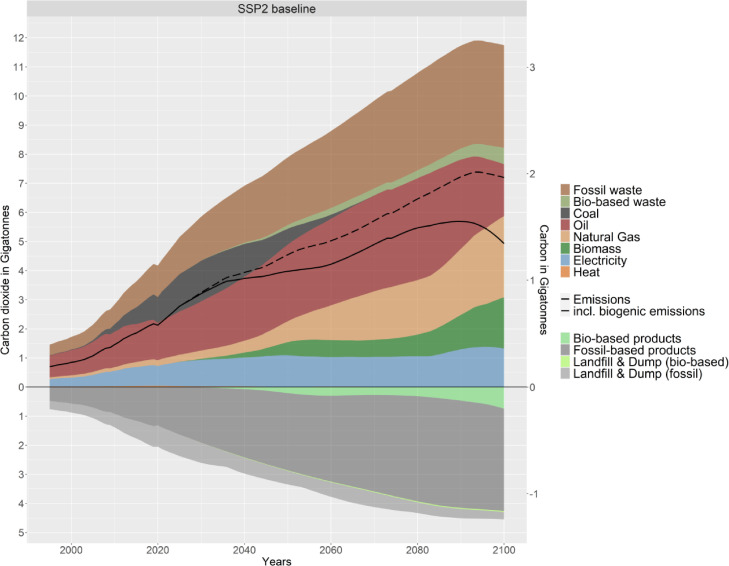
Carbon balance of the global plastics sector over the entire life cycle.

A key output of the model is the overall carbon balance of the plastics sector over its entire life-cycle. Fig. 11 shows the carbon inputs into the sector on the upper side of the graphs, including the carbon in the plastic waste generated each year that could be either used as a resource via recycling, or end up incinerated or in landfills. Furthermore, it shows all carbon sequestered in product stocks and landfills or dumps (bottom part of the graph). The lines show the overall emission balance of the plastics sector, both with and without considering biogenic emissions.

**Fig. 12:**
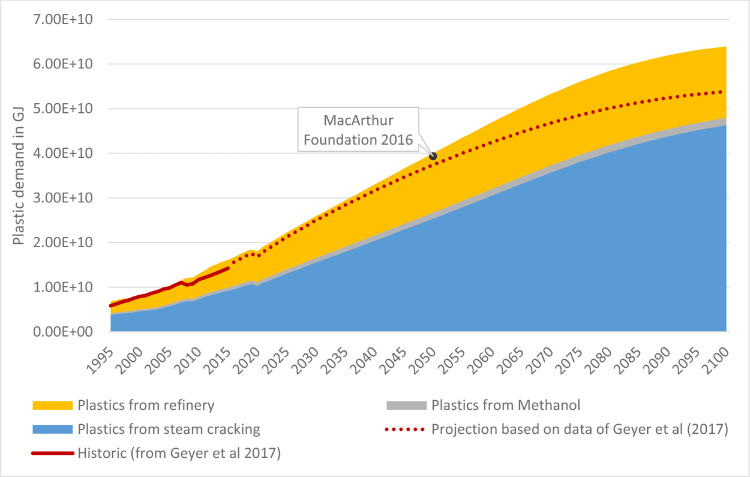
Comparison of plastic demand projections (sum of stacked lines is the PLAIA model projection).

## Discussion of limitations

With PLAIA, we created one of the first global, long-term models on plastic production and waste management as part of an IAM. The model is suitable to analyze the impact of different material use and emission mitigation strategies throughout the entire life cycle of plastics. Our efforts represent the first steps to better model non-energy use, materials, and the circular economy in IAMs. Further improvements are necessary to tackle the limitations of our work.

A fundamental issue was the limited data available for the chemical sector [31]. Also, it is a challenge to find useful data for downstream plastic production and waste management, particularly when aiming to represent the 26 world regions in IMAGE. This lack of data forced us to accept several limitations in our model. We summarize the key limitations and suggestions for improvements in this section.

### Modeling the demand for plastics

In the absence of actual demand data, we assumed historical production data to represent demand. Moreover, we chose to model plastic demand based on the upstream production of intermediate chemicals used for plastics as no regionally disaggregated plastic production data was available.

To have a benchmark for our approach of modeling plastic demand, we also created a plastic demand projection based on the global plastic production data of Geyer et al. [18]. We assumed a logistic growth relationship between plastic production per capita and GDP per capita (same as Eq. 1, but with an Alpha of 7.51 and a Beta of 14525). Fig. 7 compares historical data and the projection of global plastic demand based on the data of Geyer et al. [18] with the demand implemented in the model (as a function of HVC, refinery products, and methanol) for an SSP2 GDP/cap projection.

PLAIA produces similar results to historical values from Geyer et al. [18], even though they differ regarding the dip in historical plastic demand after the financial crisis of 2008. The projections until 2050 are broadly similar, with differences in the range of 4-7% in this period. After 2050, the demand function implemented in PLAIA increases further before leveling off towards the end of the century, while the projections based on Geyer et al. level off at a lower value. For the year 2050, our demand projection based on upstream chemical production is almost the same as the plastic demand projection of the Ellen MacArthur Foundation [61], while the projection based on data of Geyer et al is 7% lower. Therefore, we conclude that our approach of modeling plastic demand as a function of upstream chemical production seems justifiable, especially given the advantage of allowing for a regional disaggregation of plastic demand. However, while our approach produced similar results on a global level as projections based on plastic production data, this might be different on a regional level.

The data that was used for our projections only represents primary plastic production. Therefore, the projections slightly underestimate the total plastic production as they exclude recycled plastics. Recycling of plastics was negligible until 1980 [18]. Since the 1980s, recycling rates have increased significantly, but the actual amount of recycled plastics on the market is uncertain, and there is limited publicly available information. The world petrochemicals balance of 2004 from Gielen et al. [19] estimates that around 4% of plastics originate from recycling. A more recent study by PlasticsEurope [43] concluded that in 2018 around 4 million tonnes of plastics produced in the European Union were from recycled materials, which equals around 6% of its production by that year. This number is from a high recycling region, which sends approximately 32% of its plastic waste to recycling [43]; we expect much lower percentages of recycled plastics globally. Furthermore, recycling rates were much lower in the past, meaning that its market share in historical plastic production is likely to be marginal. Therefore, we consider demand derived from primary production data alone to be a sufficiently accurate indicator for plastic demand.

PLAIA does not include the trade of chemicals and plastic (waste). Hence, PLAIA assumes that plastics are consumed and that waste is treated in the producing region. This assumption might lead to underestimating plastic consumption in regions with historically limited chemical production, i.e., developing economies, and overestimating it in regions with high chemical production. In conclusion, our model would benefit from country-specific plastic consumption data and the inclusion of trade. The latter is a challenge because it is difficult to follow trade flows through all life cycle stages of plastics: intermediate chemicals, plastic polymers, plastic products, and finally, plastic waste.

### Waste collection

We model waste collection based on GDP/capita development. Since our model assumes that waste is generated and collected in the producing region (see the previous section), we give more weight to the high collection rates in developed economies, probably leading to overestimating global plastic waste collection.

Moreover, modeling waste collection as a function of GDP/cap is a simplification, ignoring other factors driving collection rates such as policy developments and urbanization. In general, reliable data for collection rates is difficult to obtain for many regions [51]. In many low to upper-middle-income countries (World Bank definition), informal waste systems play a vital role whose contributions are difficult to measure. Furthermore, the World Bank data refers to overall waste collection rates and is thus ignoring differences between specific waste streams such as plastics.

### Data bias, omissions, and simplified assumptions

We predominately used technology and cost data from Europe and the USA due to data limitations for other regions. Only endogenously modeled inputs like the energy mix and cost as well as the land price are region-specific. The model could achieve a better regional representation of plastic production and waste management with more country-specific data sources.

Some steps in the life cycle of plastics have a simplified representation or are missing. PLAIA uses proxies and aggregated data on many occasions. While this practice reduces the granularity of the model, it is common practice for an IAM that deals with aggregated, global & long-term developments.

#### Limitations in the upstream chemical production

The production of chemicals from steam crackers is modeled in detail, covering various fossil and bio-based feedstocks and eight conversion routes. Steam cracking provides ca 65% of monomer inputs in plastics production [31]. The modeling of methanol production is less detailed but still includes estimates for process energy and transformation losses. The refinery is the most opaque source of olefins and aromatics used in plastics [31] and thus the most difficult to cover. Daioglou et al. [7] modeled refinery products in an aggregated, simplified way. Due to missing data, the model only provides the net energy bound in refinery products and thus ignores process energy and transformation losses.

In the absence of continuous historical data, we assume constant shares of steam cracking products, refinery sourced olefins & aromatics, and methanol as feedstock for plastics (see chapter 3.2). While this simplification ignores future changes in production pathways, it can, to a certain extent, be supported by the observation that the chemical sector seems to be relatively constant when it comes to its production pathways and end-uses. Levi and Cullen [31] compared their results for 2013 with two other studies with data of 2004 and 2006. This comparison showed that the shares between the end-use sectors were comparable to the 2013 data of Levi and Cullen [31]. However, fundamental changes in the chemical sector might change the sources of plastic feedstocks in the future.

#### Limitations in the downstream plastic production

We created proxies for plastic polymerization and transformation based on weighted averages of current data. This practice ignores potential changes in plastic types and shares of technology routes in the future. Similarly, assuming a constant LHV for plastics is a simplification and ignores future changes in the market shares and the introduction of new, different plastic types. New plastics, particularly biobased ones, could have other chemical structures and thus different LHVs and carbon contents. Currently, biobased plastics represent around 1% of the global market (European Bioplastics e.V. (EUBP) [11]). So far, the majority of the biobased plastics on the market are drop-in plastics, meaning they have the same chemical structure and LHV as their fossil competitors (e.g., bio-PET) (European Bioplastics e.V. (EUBP) [11]). But there are also upcoming plastic types with different structures and lower LHVs, like Polylactic acid (PLA) or Polyethylene Furanoate (PEF). Furthermore, there are also discussions about producing plastics from captured CO_2_ in the future [27]. Changes in the chemical structures of plastics could influence our model results in several ways: E.g., a lower LHV would make waste to energy a less attractive solution, while a lower carbon content would reduce the GHG emissions of incinerating plastics. However, it is highly speculative to forecast the future market shares of these new plastic types and how far they would change the average LHV of plastics. Therefore, we chose to keep the simplified assumption of a constant average LHV of plastics.

#### Limitations in waste management

In cases where data was unavailable or unreliable, we chose to leave it out rather than include it based on uncertain assumptions. This was the case for the collection and transportation of plastic waste, as it is difficult to find representative data for all IMAGE regions on average transportation distances. The impact of this omission on the overall GHG emissions of plastics is marginal but not negligible. For example, Hestin et al. [23] showed that in the European Union, the collection and transportation steps cause around 3-4% and 4-5 % of the GHG emissions of recycling, respectively, depending on the plastic type.

However, collection can have a significant share in total recycling costs [51]. But as all waste treatment options require waste collection & transportation, these costs have a limited impact on the relative costs of the waste treatment options to each other. However, there are differences in transportation needs between the options, depending on the region (e.g., distances to the closest recycling center or landfill; transport of recycling rejects to landfill or incineration plant). Therefore, ignoring collection & transport is still a limitation of this model.

Next to mechanical recycling, we only include chemical recycling via pyrolysis as an alternative recycling route. There are many other ways of chemically recycling plastic waste. While pyrolysis recycles plastic back to its feedstock, other technologies allow recycling back to monomer. However, these promising routes are still in a research & development stage or are operating on a small scale, with very little public data available. Furthermore, these routes are polymer-specific and mostly require a very pure input stream. Since this model covers plastics in an aggregated way and data availability is scarce, polymer-specific chemical recycling routes are not part of the model yet. Also other plastic waste treatment methods like gasification or emerging technologies like photoreforming are not yet included [63]. With increasing data availability, the model will be updated to include further promising plastic waste treatment methods.

### Technological learning

IMAGE and TIMER include technological improvements via learning for the production of energy carriers [53]. In particular, biomass production routes benefit from learning [7]. However, in the current model version, PLAIA does not include technological learning for waste treatment technologies, even though future gains are likely. Already today, sorting and recycling yields can reach higher values for specific sectors or plastic types. Sorting yields for packaging and construction plastic waste could be more than 80%, while other sectors like electronics only reach 50% [23]. Recycling yields for PET plastics could reach more than 90% and are thus significantly higher compared to other plastic types [14].

It is difficult to make sound projections on the development of the recycling sector. The most significant potential improvements are not technology but policy-related. Fostering circular product design and enforcing a better sorting of plastics (e.g., via deposit systems) will likely have a much more significant impact than technical improvements in sorting and recycling machinery. Moreover, we would require data on historical improvements to define technological learning rates for recycling technologies. So far, we kept the assumptions stable over time for the baseline scenario (apart from the endogenous variable costs such as energy prices). However, we will simulate developments towards a circular economy and resulting yield and cost improvements via scenarios in upcoming publications. Nevertheless, future updates regarding technological learning are desirable to improve the PLAIA model further. To achieve this, additional research on technological learning rates for plastic production, mechanical recycling, chemical recycling, and incineration technologies would be required.

### Limitations in carbon accounting

Globally the CO_2_ emissions of the chemical sector sum up to around 1.5 Gt of CO_2_ emissions a year (18% of global industrial CO_2_ emissions) [26]. Additionally, the sector produces globally non-CO_2_ greenhouse gas emissions in the range of 350-400 million tonnes in CO_2_ equivalents a year, mainly consisting of hydrofluorocarbons (HFC) and nitrous oxide [15]. Some of these emissions can be attributed to plastics; for example, the nitrous oxide emissions from adipic acid production used in nylons [15] or the HFC emissions from blowing agents used in producing extruded-polystyrene and polyurethane foams [20]. These additional non-CO_2_ emissions are not accounted for in the model.

To achieve an inherently consistent carbon balance, we calculate the carbon content of plastics based on the shares of the feedstock energy carriers used for producing plastics. However, this approach ignores the chemical transformations from feedstock to product: Not all feedstock carbon might end up in the product. We currently lack the necessary data to include these transformations in the model.

An alternative way would be to use a fixed carbon content that is constant over time and regions. For example, the IPCC emission factor database offers emission factors for plastics [9,25]. Using this exogenously set carbon content leads to inconsistent carbon balances since they do not relate to the feedstock inputs into the model. In extreme cases, when using high amounts of low-carbon feedstock like natural gas, this fixed carbon content could lead to a negative balance, meaning that more carbon is assumed to be sequestered than what went into the production. Furthermore, these exogenous values are averages and differ by the reporting country.

Both approaches are essentially incorrect. To guarantee consistency within the model, we chose to define the carbon content of plastics based on the shares of the feedstock energy carriers used for their production. Compared to a fixed carbon content for plastics based on the IPCC factor^4^, we see that the endogenously modeled weighted global average carbon content is 5-15% lower, varying per year.

We assume that plastic carbon going to landfills and dumps stays sequestered for the entire analyzed period (up to 2100). The key reason for this choice was the limited data on the degradation of plastics in different environments and its implications on Greenhouse-gas emissions [4,48]. However, plastic seems to have a very slow chemical degradation rate when buried in landfills: A review of Chamas et al. [4] showed a half-life of hundreds or thousands of years for most plastics (i.e., HDPE, PVC, PET, PS) when buried. Only LDPE bags showed a short half-life of 4.6 years.

However, the degradation speeds up when the plastics are submitted to sunlight or in marine environments [4,48]. Therefore, one might consider emissions in particular of the openly dumped plastics. Unfortunately, there is not yet sufficient literature to base an emission factor on. Such an emission factor depends on plastic types, the environment they are in, their exposure to sun, heat, and oxygen, and their surface [4,48]. Initial assessments indicate that emissions of dumped plastic waste play a minor role in the global GHGeq budget [48]. Furthermore, the dumping of plastics only plays a small role in the PLAIA model due to the reasons mentioned previously.

### Recommendations for further research

This section outlined some of the key limitations we faced when developing the PLAIA model. The major reason for the limitations lies in the data: The collection and provision of data on the chemical, plastic, and waste management sectors need to improve significantly to provide a better basis for research and policies. Model improvements should focus on including trade, technological learning, and a better representation of regional specifics in technologies, costs, and policies (particularly for China, a rapidly growing plastic producer). Moreover, a better link of the PLAIA model to other industry sectors would allow for a truly integrated assessment. It would allow for using wastes from other industry sectors as a resource for chemical production (e.g., black liquor from the pulp & paper industry) and for modeling the competition of plastic materials with alternatives for specific applications (e.g., packaging made of plastics or paper/cardboard). This initial version of the PLAIA model can just be seen as the first step to better integrating materials and the circular economy in IAMs and requires continuous improvement. However, despite its limitations, PLAIA can already provide valuable insights into the impact of different material use and emission mitigation strategies throughout the entire life cycle of plastics.

## Declaration of Competing Interest

The authors declare that they have no known competing financial interests or personal relationships that could have appeared to influence the work reported in this paper.

## Appendix Supplementary materials

Image, application 1

## Footnotes

1 Defined in NEDE as the outputs of steam cracking, which are the intermediate building blocks of the organic petrochemical industry (ethylene, propylene, butadiene, aromatics) [7]

2 Representing the heavier refinery products coming from the distillation of crude oil. Refinery products in NEDE consist of lubricants, aromatics and bitumen [7].

3 See e.g. S&P Global's comparison of ethylene vs LDPE and propylene vs PP price comparison of averages from November 2017 to March 2018: Ethylene and propylene prices ranges between 0.75 and 0.85 of LDPE and PP prices [49].

4 Carbon weight = 75% of plastic weight. This would result in 21,42 kg Carbon/GJ plastic (assuming an average plastic LHV of 35 GJ/t of plastics)
