# Supplementary material for: The plastics integrated assessment model (PLAIA): Assessing emission mitigation pathways and circular economy strategies for the plastics sector
Source: MethodsX. 2022 Mar 15;9:101666. doi: 10.1016/j.mex.2022.101666 (PMC8965151; doi:10.1016/j.mex.2022.101666)
Supplement: Supplementary file 1 [file mmc1.pdf]

Supplementary materials

These supplementary materials complement the following article:

Stegmann, P., Daioglou, V., Londo, M. & Junginger, M. The plastics integrated assessment model (PLAIA): Assessing emission mitigation pathways and circular economy strategies for the plastic sector. MethodsX (2022). https://doi.org/10.1016/j.mex.2022.101666.

Figure 1: The IMAGE framework region classification (PBL 2018)

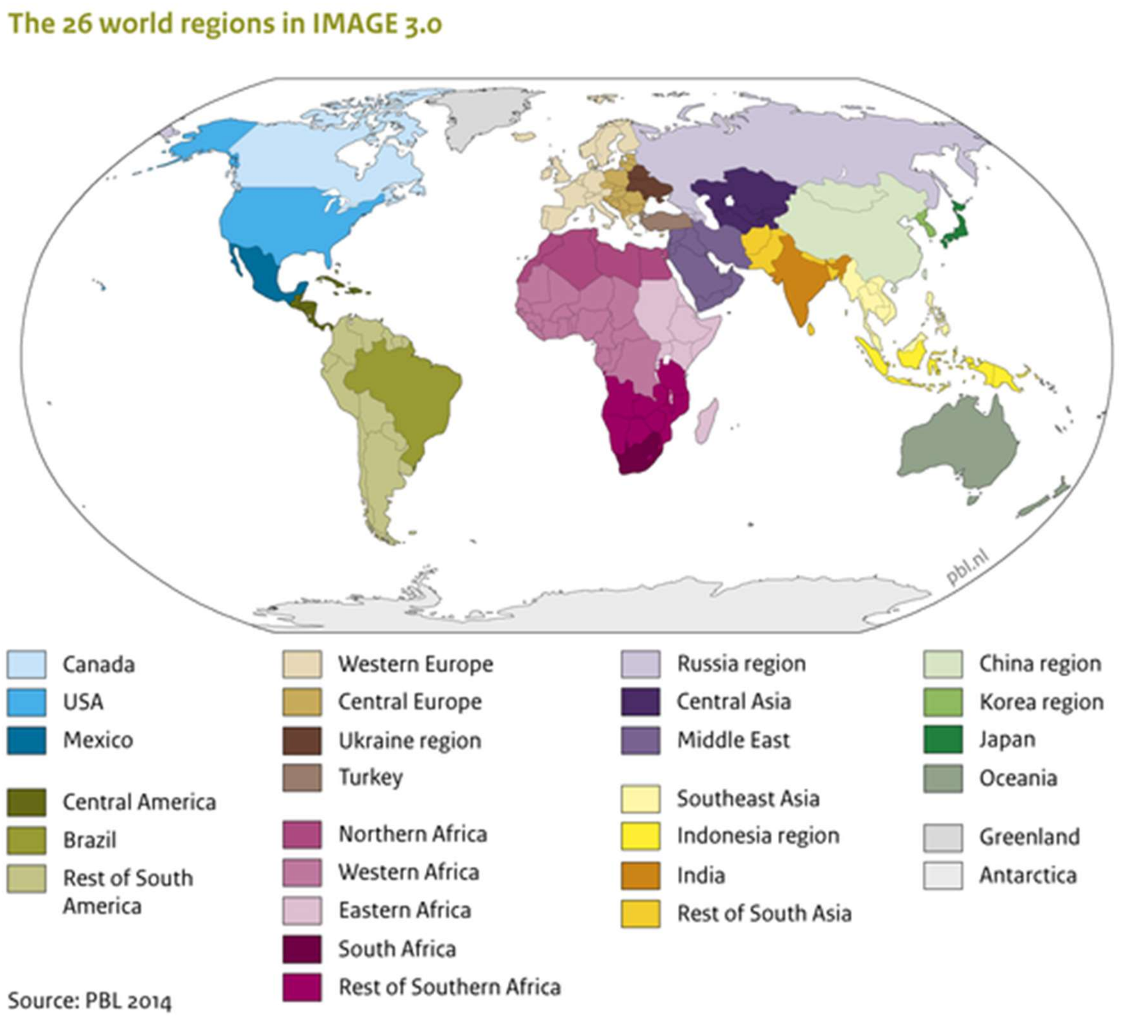

Table 1: Classification of IMAGE regions

Region	Nr	Countries
Canada	1	Canada (124)
USA	2	St. Pierre and Miquelon (666), United States (840)
Mexico	3	Mexico (484)
Central America	4	Anguilla (660), Aruba (533), Bahamas, The (44), Barbados (52), Belize (84), Bermuda (60), Cayman Islands (136), Costa Rica (188), Dominica (212), Dominican Republic (214), El Salvador (222), Grenada (308), Guadeloupe (312), Guatemala (320), Haiti (332), Honduras (340), Jamaica (388), Martinique (474), Montserrat (500), Netherlands Antilles (530), Nicaragua (558), Panama (591), Puerto Rico (630), St. Kitts and Nevis (659), St. Lucia (662), St. Vincent and the Grenadines (670), Trinidad and Tobago (780), Turks and Caicos Isl. (796), Virgin Isl. (Br.) (92), Virgin Islands (U.S.) (850)
Brazil	5	Brazil (76)
Rest of South America	6	Argentina (32), Bolivia (68), Chile (152), Colombia (170), Ecuador (218), Falklands Isl. (238), French Guyana (254), Guyana (328), Paraguay (600), Peru (604), Suriname (740), Uruguay (858), Venezuela, RB (862)
Northern Africa	7	Algeria (12), Egypt, Arab Rep. (818), Libya (434), Morocco (504), Tunisia (788), Western Sahara (732)
Western Africa	8	Benin (204), Burkina Faso (854), Cameroon (120), Cape Verde (132), Central African Republic (140), Chad (148), Congo, Dem. Rep. (180), Congo, Rep. (178), Cote d'Ivoire (384), Equatorial Guinea (226), Gabon (266), Gambia, The (270), Ghana (288), Guinea (324), Guinea-Bissau (624), Liberia (430), Mali (466), Mauritania (478), Niger (562), Nigeria (566), Sao Tome and Principe (678), Senegal (686), Sierra Leone (694), St. Helena (654), Togo (768)
Eastern Africa	9	Burundi (108), Comoros (174), Djibouti (262), Eritrea (232), Ethiopia (231), Kenya (404), Madagascar (450), Mauritius (480), Reunion (638), Rwanda (646), Seychelles (690), Somalia (706), Sudan (736), Uganda (800)
South Africa	10	South Africa (710)
Western Europe	11	Andorra (20), Austria (40), Belgium (56), Denmark (208), Faeroe Islands (234), Finland (246), France (250), Germany (276), Gibraltar (292), Greece (300), Iceland (352), Ireland (372), Italy (380), Liechtenstein (438), Luxembourg (442), Malta (470), Monaco (492), Netherlands (528), Norway (578), Portugal (620), San Marino (674), Spain (724), Sweden (752), Switzerland (756), United Kingdom (826), Vatican City State (336)
Central Europe	12	Albania (8), Bosnia and Herzegovina (70), Bulgaria (100), Croatia (191), Cyprus (196), Czech Republic (203), Estonia (233), Hungary (348), Latvia (428), Lithuania (440), Macedonia, FYR (807), Poland (616), Romania (642), Serbia and Montenegro (891), Slovak Republic (703), Slovenia (705)
Turkey	13	Turkey (792)
Ukraine region	14	Belarus (112), Moldova (498), Ukraine (804)

Central Asia	15	Kazakhstan (398), Kyrgyz Republic (417), Tajikistan (762), Turkmenistan (795), Uzbekistan (860)
Russia region	16	Armenia (51), Azerbaijan (31), Georgia (268), Russian Federation (643)
Middle East	17	Bahrain (48), Iran, Islamic Rep. (364), Iraq (368), Israel (376), Jordan (400), Kuwait (414), Lebanon (422), Oman (512), Qatar (634), Saudi Arabia (682), Syrian Arab Republic (760), United Arab Emirates (784), Yemen, Rep. (887)
India	18	India (356)
Korea region	19	Korea, Dem. Rep. (408), Korea, Rep. (410)
China region	20	China (156), Hong Kong, China (344), Macao, China (446), Mongolia (496), Taiwan (158)
Southeastern Asia	21	Brunei (96), Cambodia (116), Lao PDR (418), Malaysia (458), Myanmar (104), Philippines (608), Singapore (702), Thailand (764), Vietnam (704)
Indonesia region	22	East Timor (626), Indonesia (360), Papua New Guinea (598)
Japan	23	Japan (392)
Oceania	24	American Samoa (16), Australia (36), Cook Isl. (184), Fiji (242), French Polynesia (258), Kiribati (296), Marshall Islands (584), Micronesia, Fed. Sts. (583), Nauru (520), New Caledonia (540), New Zealand (554), Niue (570), Northern Mariana Islands (580), Palau (585), Pitcairn (612), Samoa (882), Solomon Islands (90), Tokelau (772), Tonga (776), Tuvalu (798), Vanuatu (548), Wallis ans Futuna Island (876)
Rest of South Asia	25	Afghanistan (4), Bangladesh (50), Bhutan (64), Maldives (462), Nepal (524), Pakistan (586), Sri Lanka (144)
Rest of Southern Africa	26	Angola (24), Botswana (72), Lesotho (426), Malawi (454), Mozambique (508), Namibia (516), Swaziland (748), Tanzania (834), Zambia (894), Zimbabwe (716)

Figure 2: Historical data points for each IMAGE region, relating HVC demand/cap to GDP/cap *(except for regions without steam crackers according to Oil & Gas Journal: 4, 9, 25, 26); for number legend and region names see Table 1 above*

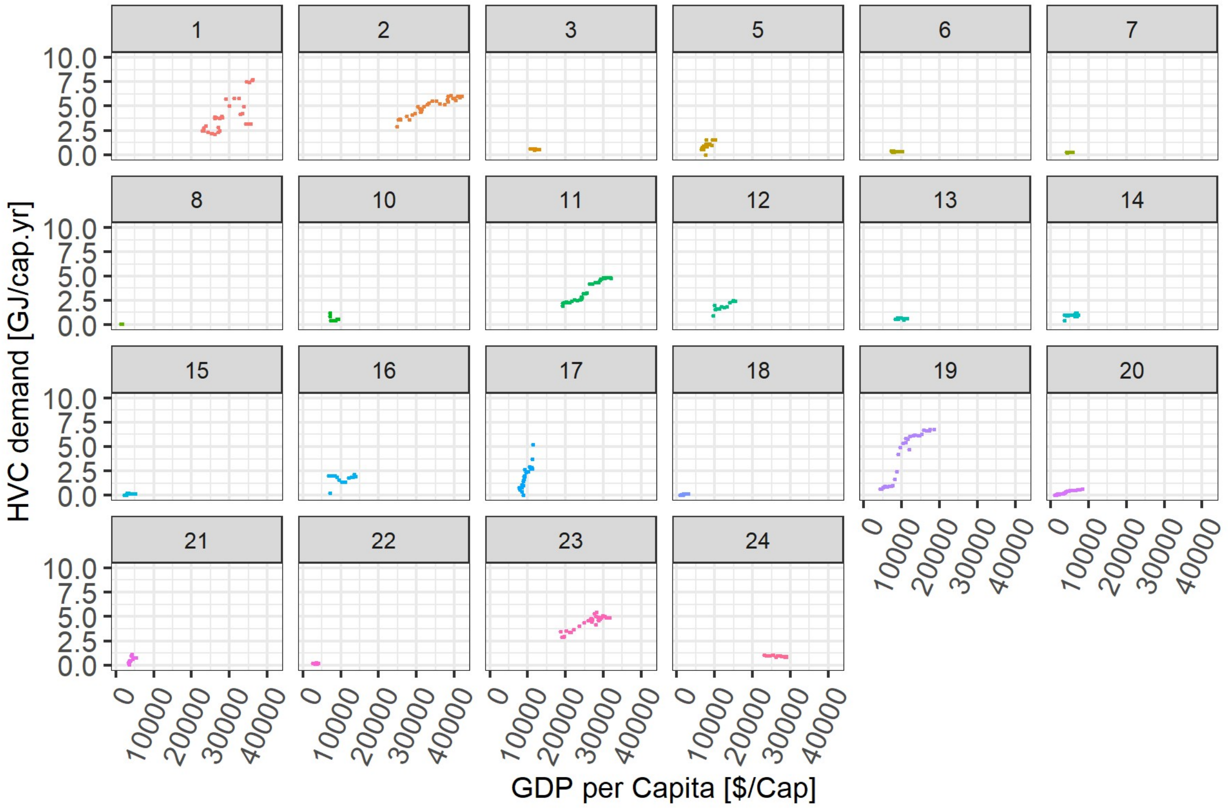

***Figure 3: The structure of the updated NEDE model**, including the additions of PLAIA. PLAIA is integrated into NEDE and models the downstream plastic production, products use, and end of life.*

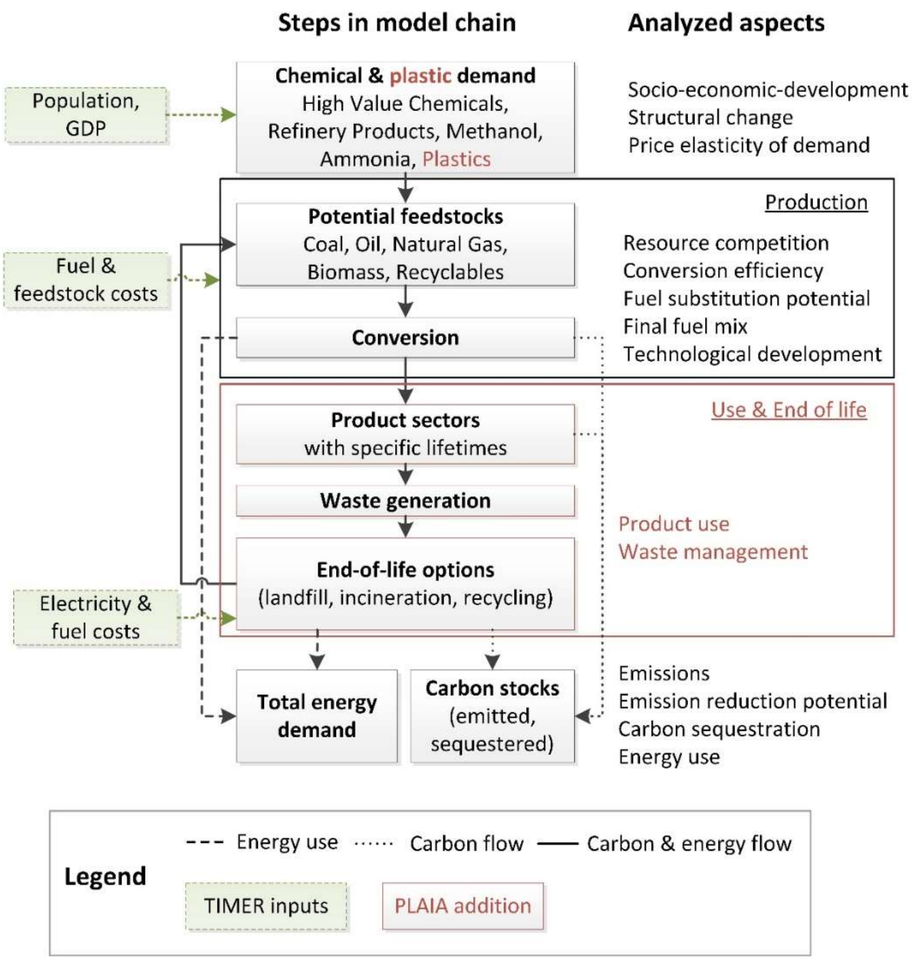

Table 2: Lower heating values of steam cracking feedstocks and products

LHV of Steam cracker feedstocks in GJ/t		LHV of Steam cracker products in GJ/t	
Ethane	47.8	Ethylene	47
Propane	46.4	Propylene	47
Butane	45.3	C4 stream	45
Naphtha	44.9	Aromatics	40
Gas Oil	42.8	Sources: Ren (2009) for ethylene & propylene; Mozaffarian (2015) for Aromatics; LHV of C4 stream is a rounded assumption based on LHVs of butadiene, butene, and Isobutene (ChemEurope 2021)	
Other	45.5		
Source: Engineering ToolBox (2003), assuming LHV of LPG for Other			

Table 3: Steam cracker yields in GJ product / GJ feedstock

Products	Feedstocks					
	Ethane	Propane	Butane	Naptha	Gas oil	Other[a]
Ethylene	0.790	0.471	0.458	0.339	0.275	
Propylene	0.016	0.127	0.157	0.176	0.158	
Butadiene	0.022	0.047	0.044	0.050	0.053	
Other C4	0.006	0.012	0.033	0.062	0.042	
Aromatics	0	0	0	0.092	0.117	
Total HVC yield	0.833	0.656	0.691	0.719	0.644	0.708

Based on Levi & Cullen (2018);
[a] Yield from feed "Other" is assumed to be the average of the other 5 yields. "Other" represents only a minor share in total feedstocks and refers to LPG, NGL, Hydrowax, Refinery gas and kerosene (Oil & Gas Journal 1997-2012).

Table 4: Regression coefficients for modeling HVC demand

IMAGE region	China+	Eastern Europe	Western Europe	Japan	Korea+	Middle East	South East Asia	USA	Rest of World
**Alpha**	7.98	13	13	10.81	13	13	9.98	12.31	7.98
**Beta**	17288.04	28645.64	35834.84	23635.1	10419.68	13710.31	17177.59	31169.06	20055.88

Table 5: Average lower heating values (LHV) per plastic type

	LD,LDPE	HDPE	PP	PS	PVC	PET	PUR	PP&A
**Average LHV in GJ/t**	43,35	41,44	43,19	40,19	19,69	23,15	26,53	24,71 – 29,81[a]

[a]depending on polyester share;
Sources: (Brunner, Mark, and Kamprath 2000; Chiellini and Solaro 2003; Franklin Associates 2011; H. Mozaffarian 2015; TNO 2020; Tsiamis and Castaldi 2016; Wasilewski and Siudyga 2013; Wittbecker, Daems, and Werther 1999)

Table 6: Energy use for plastic polymer production from monomers

**Plastic polymers**	HDPE	LDPE	LLDPE	PP	PET	PVC	PS	Weighted average[a]
**Electricity** in GJ/t polymer resin	1.56	3.43	1.27	1.27	2.26	4.93	0.59	2.13
**Heat** in GJ/t polymer resin	1.32	-0.25	0.69	0.84	8.54	9.03	1.29	2.84

[a] based on polymer market shares from Geyer et al. (2017)
Energy use data: (PlasticsEurope 2016b, 2016a, 2016c, 2017)

Table 7: Energy use & efficiency for transforming plastic resins into semi-finished plastic products

**Plastic polymers**	PE	PP	PET	PVC	Weighted average[a]
**Energy use** in GJ/t product output	12.77	7.21	7.72	5.73	9.6
**Efficiency** in t product output/ t resin input	1	0.91	0.94	0.97	0.96
[a] based on polymer market shares of Geyer et al. (2017) and assuming PE values for HDPE, LDPE & LLDPE					

Table 8: Mean use time of plastics by sector and their standard deviation *(from Geyer et al. 2017)*

**Market sector**	**Mean time of use in years**	**Standard deviation**
Packaging[a]	0,5	0.1
Transportation	13	3
Building and Construction	35	7
Electrical/ Electronic	8	2
Consumer & Institutional Products	3	1
Industrial Machinery	20	3
Textiles	5	1.5
Other	5	1.5
[a] We changed the distribution of packaging in PLAIA as the model has a yearly resolution. In PLAIA, all packaging plastics become waste after one year.		

Table 9: Waste collection rates for different national income levels

**Income Type**	**Collection rate** in %	**GNI/cap min** in 2005 USD	**GNI/cap max** in 2005 USD
High Income	96	12476	
Upper-middle income	82	4036	12475
lower-middle income	51	1026	4035
low income	39	0	1025
Source: *Silpa Kaza et al. (2018)*; translated to 2005 USD			

Table 10: Data used for modeling the waste treatment options

**Waste treatment option**	**Yield**	**Fixed cost factor**[b]	**Substitution rate**	**Electricity use**	**Heat use**	**Diesel use**
	GJ plastics / GJ plastic waste	2005 USD/ GJ plastic waste	value of recycled to primary pl.	GJ/GJ plastic waste	GJ/GJ plastic waste	GJ/GJ plastic waste
Sorting	0.75 for MR<br>0.85 for CR	4	-	0.0058	-	-
Mechanical Recycling	0.75	10	0.81	0.0527	0.0064	-
Chemical Recycling [a] (via pyrolysis)	0.315	16	1	-	0.59 (natural gas)	-
Waste to Energy	-	3.15	-	-0.09	-0.22	-
Landfilling	-	1.8	-	0.0007	-	0.002

[a] includes the production of naphtha from plastic waste & the transformation of naphtha to monomers. It excludes the polymerization step, which is added in the model as described in 4.2
[b] excludes collection & transportation and excludes energy use (heat, electricity, diesel, which are added endogenously in the model)

Data sources: (Arena, Mastellone, and Perugini 2003; Daioglou et al. 2014; European Commission 2018; Faraca, Martinez-Sanchez, and Astrup 2019; Gradus et al. 2017; Gu et al. 2017; Hestin, Faninger, and Milios 2015; Manfredi et al. 2009; Ren 2009; Rigamonti et al. 2014; Wong 2010; WRAP 2009)

References

Arena, Umberto, Maria Laura Mastellone, and Floriana Perugini. 2003. “Life Cycle Assessment of a Plastic Packaging Recycling System.” *International Journal of Life Cycle Assessment* 8(2):92–98.

Brunner, M., F. E. Mark, and A. Kamprath. 2000. *Composition of Old Car Seat Foams*. Rapperswil.

ChemEurope. 2021. “Heat of Combustion.” Retrieved October 25, 2021 (https://www.chemeurope.com/en/encyclopedia/Heat_of_combustion.html).

Chiellini, Emo and Roberto Solaro. 2003. *Biodegradable Polymers and Plastics*. New York: Springer Science+Business Media.

Daioglou, Vassilis, Andre P. C. Faaij, Deger Saygin, Martin K. Patel, Birka Wicke, and Detlef P. van Vuuren. 2014. “Energy Demand and Emissions of the Non-Energy Sector.” *Energy Environ. Sci.* 7(2):482–98.

Engineering ToolBox. 2003. “Fuels - Higher and Lower Calorific Values.” Retrieved February 25, 2020 (https://www.engineeringtoolbox.com/fuels-higher-calorific-values-d_169.html).

European Commission. 2018. *Environmental Impact Assessments of Innovative Bio-Based Product*. Luxembourg.

Faraca, Giorgia, Veronica Martinez-Sanchez, and Thomas F. Astrup. 2019. “Environmental Life Cycle Cost Assessment: Recycling of Hard Plastic Waste Collected at Danish Recycling Centres.” *Resources,*

Conservation and Recycling 143(October 2018):299–309.

Franklin Associates. 2011. *Cradle-to-Cradle Life Cycle Inventory of Nine Plastic Resins and Four Polyurethane Precursors*. Prairie Village, Kansas.

Geyer, Roland, Jenna Jambeck, and Kara Law. 2017. “Production, Use, And Fate Of All Plastics Ever Made.” *Science Advances* 3(7):25–29.

Gradus, Raymond H. J. M., Paul H. L. Nillesen, Elbert Dijkgraaf, and Rick J. van Koppen. 2017. “A Cost-Effectiveness Analysis for Incineration or Recycling of Dutch Household Plastic Waste.” *Ecological Economics* 135:22–28.

Gu, Fu, Jianfeng Guo, Wujie Zhang, Peter A. Summers, and Philip Hall. 2017. “From Waste Plastics to Industrial Raw Materials: A Life Cycle Assessment of Mechanical Plastic Recycling Practice Based on a Real-World Case Study.” *Science of the Total Environment* 601–602:1192–1207.

Hestin, Mathieu, Thibault Faninger, and Leonidas Milios. 2015. *Increased EU Plastics Recycling Targets: Environmental, Economic and Social Impact Assessment*.

Levi, Peter G. and Jonathan M. Cullen. 2018. “Mapping Global Flows of Chemicals: From Fossil Fuel Feedstocks to Chemical Products.” *Environmental Science and Technology* 52(4):1725–34.

Manfredi, Simone, Davide Tonini, Thomas H. Christensen, and Heijo Scharff. 2009. “Landfilling of Waste: Accounting of Greenhouse Gases and Global Warming Contributions.” *Waste Management and Research* 27(8):825–36.

Mozaffarian. 2015. *Market Analysis for Lignocellulosic Biomass as Feedstock for Bioenergy, Biobased Chemicals & Materials in Europe; A Quantitative Estimate of Biomass Demand in 2020 and 2030*. Amsterdam.

Mozaffarian, Hamid. 2015. *Market Analysis of Biomethane , BTX , Methanol , Hydrogen , Ethylene , and Mixed Alcohols*. Petten.

Oil & Gas Journal. 1997 -2012. *International Survey of Ethylene from Steam Crackers*. Houston.

PBL. 2018. “The 26 World Regions in IMAGE 3.0.” Retrieved December 16, 2020 (https://models.pbl.nl/image/index.php/Region_classification_map).

PlasticsEurope. 2016a. *High-Density Polyethylene (HDPE), Low-Density Polyethylene (LDPE), Linear Low-Density Polyethylene (LLDPE)*. Brussels.

PlasticsEurope. 2016b. *Polypropylene (PP)*. Brussels.

PlasticsEurope. 2016c. *Vinyl Chloride (VCM) and Polyvinyl Chloride (PVC)*. Brussels.

PlasticsEurope. 2017. *Ecoprofile: Polyethylene Terephthalate (PET) (Bottle Grade) CPME*. Brussels.

Ren, Tao. 2009. “Petrochemicals from Oil , Natural Gas , Coal and Biomass : Energy Use , Economics and Innovation.” Utrecht University.

Rigamonti, L., M. Grosso, J. Møller, V. Martinez Sanchez, S. Magnani, and T. H. Christensen. 2014. “Environmental Evaluation of Plastic Waste Management Scenarios.” *Resources, Conservation and*

Recycling 85:42–53.

Silpa Kaza, Lisa Yao, Perinaz Bhada-Tata, and Frank Van Woerden. 2018. *What a Waste 2.0 - A Global Snapshot of Solid Waste Management to 2050*. Washington DC: World Bank.

TNO. 2020. "Database for the Physico-Chemical Composition of (Treated) Lignocellulosic Biomass, Micro- and Macroalgae, Various Feedstocks for Biogas Production and Biochar." Retrieved May 8, 2019 (https://phyllis.nl/).

Tsiamis, Demetra A. and Marco J. Castaldi. 2016. *Determining Accurate Heating Values of Non-Recycled Plastics*. New York.

Wasilewski, Ryszard and Tomas Siudyga. 2013. "Energy Recovery from Waste Plastics." *Chemik* (5):441–45.

Wittbecker, W., D. Daems, and U. Werther. 1999. *Performance of Polyurethane (PUR) Building Products in Fires*. Brussels.

Wong, Chee. 2010. *A Study of Plastic Recycling Supply Chain*. Hull.

WRAP. 2009. *A Financial Assessment of Recycling Mixed Plastics in the UK*. Banbury.
